# *Legionella* effector AnkX displaces the switch II region for Rab1b phosphocholination

**DOI:** 10.1126/sciadv.aaz8041

**Published:** 2020-05-15

**Authors:** Stefan Ernst, Felix Ecker, Marietta S. Kaspers, Philipp Ochtrop, Christian Hedberg, Michael Groll, Aymelt Itzen

**Affiliations:** 1 Department of Biochemistry and Signal Transduction, University Medical Centre Hamburg-Eppendorf (UKE), Martinistr. 52, 20246 Hamburg, Germany.; 2 Center for Integrated Protein Science Munich (CIPSM), Department Chemistry, Technical University of Munich, Lichtenbergstrasse 4, 85747 Garching, Germany.; 3 Chemical Biology Department, Leibniz-Forschungsinstitut für Molekulare Pharmakologie (FMP), Robert-Rössle-Strasse 10, 13125 Berlin, Germany.; 4 Chemical Biology Center (KBC), Department of Chemistry, Umeå University, Linnaeus väg 10, 90187 Umeå, Sweden.

## Abstract

The causative agent of Legionnaires disease, *Legionella pneumophila*, translocates the phosphocholine transferase AnkX during infection and thereby posttranslationally modifies the small guanosine triphosphatase (GTPase) Rab1 with a phosphocholine moiety at S76 using cytidine diphosphate (CDP)–choline as a cosubstrate. The molecular basis for Rab1 binding and enzymatic modification have remained elusive because of lack of structural information of the low-affinity complex with AnkX. We combined thiol-reactive CDP-choline derivatives with recombinantly introduced cysteines in the AnkX active site to covalently capture the heterocomplex. The resulting crystal structure revealed that AnkX induces displacement of important regulatory elements of Rab1 by placing a β sheet into a conserved hydrophobic pocket, thereby permitting phosphocholine transfer to the active and inactive states of the GTPase. Together, the combination of chemical biology and structural analysis reveals the enzymatic mechanism of AnkX and the family of filamentation induced by cyclic adenosine monophosphate (FIC) proteins.

## Teaser

Using a chemical approach, the crystal structure of *Legionella* AnkX in complex with human Rab1b is obtained and characterized.

## INTRODUCTION

A large number of bacteria can replicate inside eukaryotic cells. A prominent example is *Legionella pneumophila*, the causative agent of Legionnaires’ disease. This particular pathogen infects phagocytes and multiplies in the intracellular environment of the host. After phagocytotic uptake, *L. pneumophila* reprograms the phagosome and installs a replicative organelle referred to as the *Legionella*-containing vacuole (LCV). This reconstruction is mediated by more than 300 different bacterial proteins that are translocated into the host cytosol via a type 4 b secretion system (T4bSS) (*1*–*4*). As a consequence, central components of the cell functions such as cytoskeleton dynamics, vesicular trafficking, and the different levels of cell signaling are affected, thereby ensuring survival and growth of the invader inside the LCV.

A considerable number of T4bSS effector proteins are directly interfering with components of intracellular vesicular trafficking, for example, by modulating the activity of the small guanosine triphosphatase (GTPase) Rab1 (*5*). Rab proteins function as molecular switches in cellular signaling and alternate between inactive guanosine diphosphate (GDP)–bound and active guanosine trisphosphate (GTP)–bound states. In general, the activity cycle of Rab proteins is controlled by guanine nucleotide exchange factors (GEFs) that stimulate the exchange of GDP for GTP- and GTPase-activating proteins (GAPs) that restore the GDP state. Effector proteins specifically recognize activated Rab proteins and communicate the activation states to downstream cellular factors (*6*).

*Legionella* secretes six different bacterial proteins that manipulate the activity of Rab1, including DrrA (also referred to as SidM) that contains a central GEF domain, LepB that can act as a GAP, and LidA that can act as a Rab effector (*7*–*9*). In addition, the activity of Rab1 is regulated by reversible posttranslational modifications (PTMs) such as AMPylation and deAMPylation by the N-terminal domain of DrrA and SidD, respectively (*10*–*12*). The *Legionella* protein AnkX uses cytidine diphosphate (CDP)–choline to covalently transfer a phosphocholine (PC) moiety to Rab1a and Rab1b (*13*). In contrast to other GTPase interaction partners, AnkX does not notably discriminate between the active and inactive Rab1 states as shown in vitro (*14*). AnkX is also able to modify itself with PC moieties, although the biological consequences of autophosphocholination remain elusive (*15*). Last, the *Legionella* protein Lem3 acts as a dephosphocholinase that hydrolytically removes PC from Rab1b and thereby restores the unmodified GTPase (*14*, *16*).

Phosphocholination is an unconventional PTM, as it has only been detected in two incidences in eukaryotes: in secreted placental peptides (*17*) and during *L. pneumophila* infection, in which Rab1 is modified by the bacterial enzyme AnkX (*16*). AnkX contains 949 amino acids and two individual structural units: The N terminus (amino acids 1 to 350) is constituted by a FIC [filamentation induced by cyclic adenosine monophosphate (AMP)] domain that contains the CDP-choline binding pocket. In addition, the amino acid region 351 to 800 forms an ankyrin repeat (AR) domain with currently unknown function (*13*, *18*). Previous work has demonstrated that the FIC domain and the first four ARs are forming a functional unit containing a minimal Rab1b phosphocholination activity in vitro (*19*). However, a purified AnkX version lacking the terminal ARs (i.e., amino acids 688 to 800) is significantly reduced in the phosphocholination rate, indicating that the AR domain may be involved in Rab1 recognition (*15*).

The biochemical and functional consequences of Rab1 modification have been analyzed recently (*14*, *20*). At the same time, the structural basis for the enzymatic reaction has only been superficially understood (*19*). Although the catalytic domains, or parts thereof, for DrrA, SidD, and AnkX have been characterized, experimental insights into the modes of enzyme-substrate interactions are missing (*10*, *19*, *21*). We therefore developed a strategy to covalently capture Rab1b with AnkX via its nucleotide cosubstrate, thus allowing us to characterize the AnkX:Rab1b:PC complex by x-ray crystallography. To this purpose, synthetic CDP-choline chloroacetamide derivatives bearing a thiol-reactive chloroacetamide function at the choline group have been combined with strategically placed cysteines in AnkX (AnkX_Cys_) in the active site of the FIC domain. Combination of AnkX_Cys_ with Rab1b and CDP-choline chloroacetamide yielded a site-specifically linked ternary complex, whose crystal structure discloses the ARs for Rab1 recognition. In addition, the molecular insights explain how AnkX can ensure the phosphocholination of Rab1 in the active (GTP) and inactive (GDP) states by using displacement of the switch II region of the GTPase.

## RESULTS

### Covalent capture of the AnkX_Cys_:PC:Rab1b complex

The previously reported crystal structure of Ank_1–484_, comprising the catalytic FIC domain and four ARs, revealed the mode of CDP-choline binding to the enzyme’s active site (*19*). However, the molecular basis of Rab1b binding and the significance of the AR domain have not been addressed so far. Because of the low affinity of AnkX for Rab1b (dissociation constant, 122 ± 23 μM) (*14*), preparative complex formation for structure determination is hampered.

**Fig. 1:**
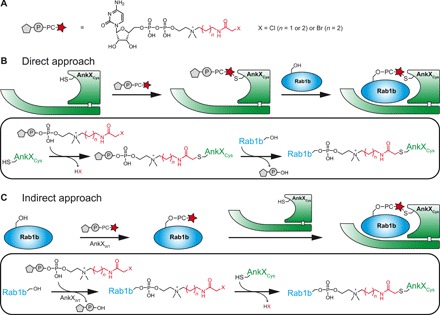
Strategy for the formation of a covalently linked AnkX_Cys_:PC:Rab1b complex. (**A**) Thiol-reactive CDP-choline analogs were prepared by attaching a chloroacetamide or bromoacetamide functionality via a short carbon spacer to the choline head group of CDP-choline. Natural CDP-choline is colored black; the synthetically installed linker and the haloacetamide group are colored red. (**B**) Direct approach. An AnkX cysteine mutant (AnkX_Cys_) is reacted with a thiol-reactive CDP-choline analog to form a binary adduct, which is then used to phosphocholinate Rab1b. (**C**) Indirect approach. Rab1b is first modified with the thiol-reactive PC group using catalytic quantities of wild-type AnkX (AnkX_WT_). The resulting Rab1b-PC conjugate subsequently forms the AnkX_Cys_:PC:Rab1b complex by the addition of stoichiometric amounts of AnkX_Cys_.

We therefore envisioned a site-specific covalent linking strategy to obtain the enzyme-PC-substrate complex for structural characterization. In particular, we intended combining thiol-reactive CDP-choline derivatives with recombinant AnkX versions equipped with strategically placed cysteines. Using our previously reported synthesis strategy (*22*), we prepared three thiol-reactive CDP-choline analogs for the site-specific capture of the AnkX_Cys_:PC:Rab1b complex (fig. S1). The nucleotides were equipped with either a moderately reactive chloroacetamide or a highly reactive bromoacetamide functionality, attached to the choline head group via a three-carbon (C3) or C4 spacer. This resulted in the successful generation of chloroacetamide-C3-CDP-choline, chloroacetamide-C4-CDP-choline, and bromoacetamide-C4-CDP-choline (referred to as C3-Cl, C4-Cl, and C4-Br, respectively) (Fig. 1A).

The use of the thiol-reactive nucleotides allows for two complementary approaches to obtaining the covalently linked AnkX_Cys_:PC:Rab1b complex. In the direct approach, AnkX_Cys_ is first conjugated with the thiol-reactive CDP-choline analog, and subsequently, this binary adduct is used for phosphocholination of Rab1b, thereby forming the covalently linked AnkX_Cys_:PC:Rab1b complex (Fig. 1B). In the alternative indirect approach, Rab1b is initially phosphocholinated with the CDP-choline analog using catalytic amounts of native AnkX [wild-type AnkX (AnkX_WT_)] (Fig. 1C). Afterward, the purified PC conjugate of Rab1b is reacted with AnkX_Cys_, thus stoichiometrically yielding the covalently linked AnkX_Cys_:PC:Rab1b complex. If not otherwise stated, then all experiments were performed using AnkX constructs including the amino acids 1 to 800 (referred to as AnkX) and Rab1b constructs including the amino acids 3 to 174 (referred to as Rab1b).

### Preparative formation of a covalently linked AnkX_Cys_:PC:Rab1b complex

**Fig. 2:**
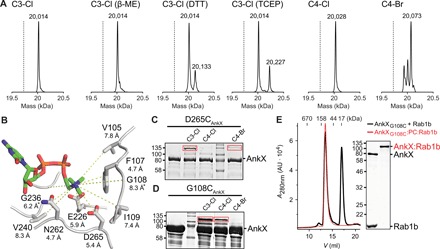
Preparative formation of a covalently linked AnkX_Cys_:PC:Rab1b complex using thiol-reactive CDP-choline analogs. (**A**) Incorporation of thiol-reactive CDP-choline derivatives into Rab1b and compatibility with different reducing agents. The calculated masses for the expected Rab1b-PC conjugates are as follows: 20,014 Da (Rab1b-PC–C3-Cl), 20,028 Da (Rab1b-PC–C4-Cl), and 20,073 Da (Rab1b-PC–C4-Br). The black dashed lines indicate the mass of unmodified Rab1b (19,729 Da). (**B**) AnkX residues selected for the mutagenesis to cysteine [Protein Data Bank (PDB) ID: 4BET] (*19*). Distances between the choline head group of CDP-choline and the γ-C atom are shown in angstrom. The α-C atom was used for glycine residues, which is marked with an asterisk (*). (**C**) Direct approach for the covalent complex formation between AnkX_D265C_ (50 μM), Rab1b (100 μM), and thiol-reactive CDP-choline derivatives (1 mM). Covalent complex formation was assessed by SDS–polyacrylamide gel electrophoresis (SDS-PAGE) gel shift assay. The red rectangles indicate the bands for the specific AnkX_D265C_:PC:Rab1b complex. (**D**) Indirect approach for the covalent complex formation between AnkX_G108C_ (200 μM), Rab1b (50 μM), and thiol-reactive CDP-choline derivatives (1 mM). Covalent complex formation was assessed by SDS-PAGE gel shift assay. The red rectangles indicate the bands for the specific AnkX_G108C_:PC:Rab1b complexes. (**E**) Analytical size exclusion chromatography of the binary AnkX_G108C_:PC:Rab1b complex. The SDS-PAGE gel shows the input of the size exclusion chromatography. *A*_280nm_, absorbance at 280 nm; AU, arbitrary units.

With a series of thiol-reactive CDP-choline analogs in hand, we attempted to produce the AnkX_Cys_:PC:Rab1b complex. First, we investigated the compatibility of the nucleotides with Rab1b phosphocholination catalyzed by AnkX_WT_. Catalytic quantities of AnkX_WT_ successfully phosphocholinated Rab1b with CDP-choline derivatives (C3-Cl, C4-Cl, and C4-Br), as indicated by the change in molecular weight using mass spectrometry (Fig. 2A). In the case of C4-Br, however, two side products were detected, which may result from the increased thiol reactivity of bromoacetamides compared to chloroacetamides, thus suggesting the bromoacetamide derivative (C4-Br) to be too reactive.

The formation of the covalent AnkX_Cys_:PC:Rab1b complex requires reducing conditions to activate the strategically placed cysteine residue for the reaction with the haloacetamides. Therefore, we assessed the compatibility of different reducing agents to identify potentially undesired covalent side products (Fig. 2A). The CDP-choline derivative C3-Cl is fully compatible with β-mercaptoethanol (β-ME), whereas 1,4-dithiothreitol (DTT) and tris(2-carboxyethyl)phosphine cross-react with the chloroacetamide, as observed by a corresponding mass shift of Rab1b-PC. Therefore, β-ME was used as reducing agent for further experiments.

Next, we aimed to identify potential cysteine substitution sites in AnkX that would allow for the covalent attachment of CDP-choline derivatives. On the basis of the previously reported crystal structure of AnkX_1–484_ in complex with CDP-choline [Protein Data Bank (PDB) ID: 4BET] (*19*), we identified nine positions located around the choline group within a distance that could potentially be bridged by the short carbon linker (C3/C4) and the haloacetamide group of the CDP-choline derivatives (Fig. 2B). Initially, we assessed the ability of AnkX_Cys_ variants to form the covalent AnkX_Cys_:PC:Rab1b complex using the direct approach by detecting the change in molecular weight SDS–polyacrylamide gel electrophoresis (SDS-PAGE) (Fig. 2C and fig. S2A). The direct approach unexpectedly generated several new bands at high molecular weight, indicating a side reaction with one of the eight endogenous cysteine residues of AnkX (referred to as unspecific AnkX_Cys_:PC:Rab1b complex). Nevertheless, for the AnkX cysteine substitutions V105C, F107C, G108C, E226C, G236C, N262C, and D265C, a specific molecular weight shift was observed for the direct approach, with the most prominent band for AnkX_D265C_ in combination with C3-Cl (Fig. 2C). Since the formation of this covalent complex was dependent on linker length and the thiol-reactive functionality of the applied CDP-choline derivative, it was concluded that the corresponding band contains an AnkX_Cys_:PC:Rab1b complex where Rab1b is covalently linked to the recombinantly introduced cysteine of AnkX_Cys_. Next, we investigated the ability of the different AnkX_Cys_ variants to form a complex with Rab1b via the indirect approach (Fig. 2D and fig. S2B). Again, all of the AnkX_Cys_ variants produced unspecific AnkX_Cys_:PC:Rab1b complexes. The specific AnkX_Cys_:PC:Rab1b complex was only observed for the G108C and G236C sites of AnkX_Cys_, with the most distinct band resulting from the combination of AnkX_G108C_ with C3-Cl. Thus, in both of the approaches, covalent complex formation is more specific using the C3-linked chloroacetamide in comparison to the C4-linked chloro- and bromoacetamide.

Encouraged by these initial results, we decided to optimize the direct approach for AnkX_D265C_ and the indirect approach for AnkX_G108C_ in combination with C3-Cl, aiming to increase the complex yield and to prevent the formation of unspecific adducts.

1) For the direct approach, complex formation between AnkX_D265C_ and C3-Cl took place at various temperatures (20° to 37°C), and yields constantly increased in time up to 72 hours (fig. S3A). Maximal binary complex formation with minimal precipitation was observed at 30°C after 72-hour incubation. In parallel, we optimized the conditions for the formation of the ternary AnkX_D265C_:PC:Rab1b complex. To this purpose, we produced the AnkX_D265C_-C3-Cl conjugate and subsequently quantified the ternary complex formation by SDS-PAGE after incubation with Rab1b. The desired ternary complex is rapidly formed at various temperatures (20° to 37°C) (fig. S3B). The total yield of the complex was limited by the amount of AnkX_D265C_-C3-Cl, indicating that the initial conjugation is critical for the preparative complex formation. The formation of unspecific AnkX_Cys_:PC:Rab1b complexes could be prevented by removing the excess CDP-choline chloroacetamide (C3-Cl) via buffer exchange from the binary complex between AnkX_D265C_ and C3-Cl before the addition of Rab1b (fig. S3, A and B).

2) For the indirect approach, formation of unspecific AnkX_Cys_:PC:Rab1b complexes could not be prevented by any additional purification steps. However, an AnkX_G108C_ mutant, in which three other cysteines C48, C84, and C172 had been replaced by serine, did not show unspecific reactions and was used for further experiments (fig. S3C). Since the production of the binary Rab1b-PC–C3-Cl adduct is quantitative (Fig. 2A), we assessed the formation of the covalent ternary complex with AnkX_G108C_ in a time-dependent manner (fig. S3C). Covalent formation slowly increased over 48 hours, showing that in the indirect approach, the reaction between AnkX_G108C_ and the Rab1b-PC–C3-Cl binary complex is rate limiting.

Together, the indirect approach produced higher amounts of ternary complex, with yields up to 60%. The covalent linking procedure could alter protein properties and lead to the formation of complex multimers. We therefore performed analytical size exclusion chromatography to determine the integrity of the produced complex (Fig. 2E). As a result, no shift in elution of AnkX_G108C_ and the covalent AnkX_G108C_:PC:Rab1b complex was observed, demonstrating the homogeneity of the preparative ternary complex. Although the covalent coupling of Rab1b with AnkX_G108C_ leads to an increase in molecular weight by ca. 20 kDa, AnkX_G108C_ and the covalent AnkX_G108C_:PC:Rab1b complex elute identically, possibly indicating that Rab1b is placed inside a preformed binding pocket.

### Structure of the AnkX-Rab1b complex

**Fig. 3:**
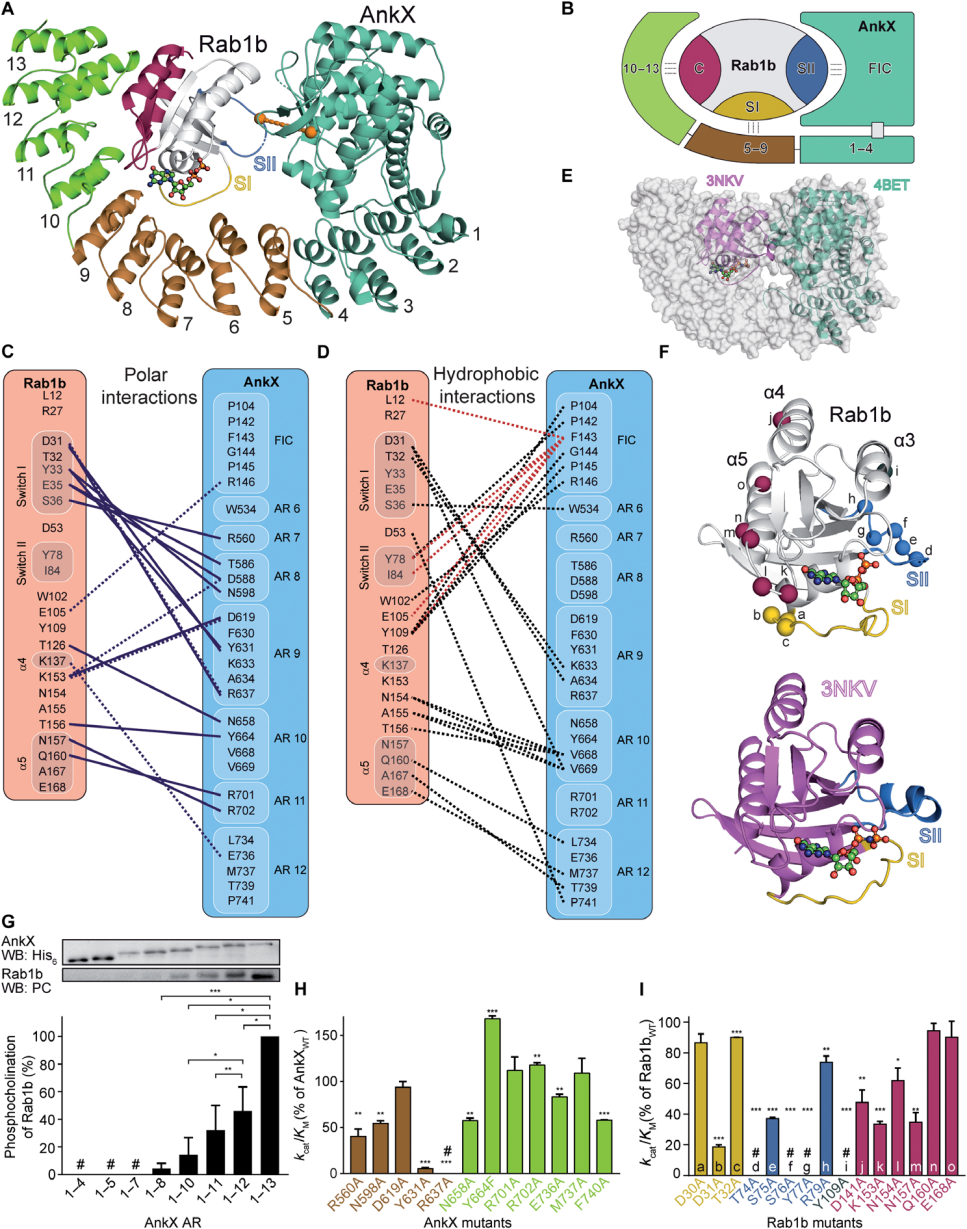
Structure of the AnkX_G108C_:PC:Rab1b:GDP complex. (**A**) Cartoon representation of the AnkX_G108C_:PC:Rab1b:GDP complex. Dark green, AnkX FIC domain and AnkX ARs 1 to 4; brown, AnkX ARs 5 to 9; light green, AnkX ARs 10 to 13; gold, Rab1b switch I (SI); blue, Rab1b switch II (SII); red, Rab1b C terminus; orange spheres, G108C_AnkX_ and S76_Rab1b_. The dashed orange line indicates the covalent linkage between G108C_AnkX_ and S76_Rab1b_. GDP is represented as a balls-and-sticks model. (**B**) Schematic model of the AnkX-Rab1b binding interface. Supposed interaction partners are the AnkX FIC domain and Rab1b switch II, AnkX ARs 5 to 9, and Rab1b switch I, as well as AnkX ARs 10 to 13 and the Rab1b C terminus. (**C**) Schematic representation of polar interactions between Rab1b and AnkX. Dashed lines, salt bridges. (**D**) Schematic representation of hydrophobic interactions between Rab1b and AnkX. Red lines indicate contacts between F143_AnkX_ to Rab1b residues. (**E**) Surface model of the AnkX_G108C_:PC:Rab1b:GDP complex. The complex structure is superimposed with active Rab1b (PDB ID: 3NKV) (*10*) and AnkX_1–484_ (PDB ID: 4BET) (*19*). (**F**) Structural evaluation of Rab1b upon binding to AnkX. Gold, Rab1b switch I; blue, Rab1b switch II; gray, α3 of Rab1b; red, Rab1b C terminus (α4 und α5). Active Rab1b bound to the nonhydrolyzable GTP-analog GppNHp (PDB ID: 3NKV) (*10*) represents the structure of the small GTPase before AnkX binding. Note that major structural rearrangements take place within the switch II region of Rab1b. (**G**) Activity assay for AnkX AR truncations. Rab1b (5 μM) was modified with CDP-choline (1 mM) by lysates of overexpressing AnkX AR truncations [total lysate (2 mg/ml)] for 2 hours, and a Western blot (WB) using an α-PC antibody and α-His antibody was performed. The signal of Rab1b phosphocholination was normalized against the signal of the AnkX His_6_-tag. (**H**) Activity assay for AnkX alanine substitutions. *k*_cat_/*K*_M_ values have been determined from phosphocholination progress curves using the change in Rab1b tryptophane fluorescence. Rab1b (5 μM) was modified with CDP-choline (1 mM) by catalytic amounts of AnkX (50 nM). (**I**) Catalytic efficiencies (*k*_cat_/*K*_M_) of Rab1b Ala mutants within the AnkX-Rab1b binding interface determined with a time-resolved tryptophane fluorescence–based assay and were normalized to wild-type Rab1b (Rab1b_WT_). Rab1b (5 μM) was modified with CDP-choline (1 mM) by catalytic amounts of AnkX (100 or 250 nM for T74A_Rab1b_, S76A_Rab1b_, Y77A_Rab1b_, and Y109A_Rab1b_). Since phosphocholination of Y109A_Rab1b_ did not result in a change of tryptophane fluorescence, the catalytic efficiency of this alanine mutant was analyzed with mass spectrometry and estimated to be of similar scale as the catalytic efficiency of T74A_Rab1b_ (fig. S8). Golden bars, Rab1b switch I; blue bars, Rab1b switch II; gray bar, α3 of Rab1b; red bars, Rab1b C terminus. Number sign (#) indicates not determined because of inactivity. *P < 0.05, **P < 0.01, and ***P < 0.001.

With the preparative complex in hand, we determined the x-ray crystal structure of the covalent ternary AnkX_G108C_:PC:Rab1b:GDP complex (3.2 Å resolution; *R*_free_ = 28.8%; PDB ID: 6SKU) by molecular replacement using the coordinates of Rab1b (PDB ID: 3NKV) (*10*) and AnkX_1–484_ (PDB ID: 4BET) (*19*) as Patterson search models (Fig. 3A and table S1). AnkX_1–800_ reveals a scoop-like structure that consists of the globular FIC domain (amino acids 1 to 350) and 13 curved ARs (amino acids 351 to 800). Thus, AnkX is forming a cavity in whose center Rab1b is embraced (Fig. 3A). Whereas ARs 1 to 4 are serving as structural support for the FIC domain as reported previously (*19*), ARs 5 to 13 are forming multiple interactions to Rab1b (Fig. 3, B to D). Rab1b shows the typical GTPase fold consisting of a central six-stranded β sheet (β1 to β6) surrounded by five α helices (α1 to α5) (Fig. 3A). The interface between AnkX and Rab1b contains fewer hydrophobic than polar interactions, which presumably contributes to the overall low affinity of the complex (*14*).

In Rab1b, no electron density is observed for the amino acids 68 to 74 of the switch II region (amino acids 64 to 83). Although the modified S76_Rab1b_ is ordered in the complex structure, no electron density is observed for PC and the linker between PC and G108C_AnkX_. This indicates the linker to be a flexible element, not forcing the covalent AnkX_G108C_:PC:Rab1b:GDP complex into any predetermined conformation. SDS-PAGE analysis of AnkX_G108C_:PC:Rab1b:GDP crystals demonstrated that the covalent linkage is still intact even after prolonged incubation at 20°C, thus excluding the possibility that the covalent complex is degraded as the result of crystallization (fig. S3D). Furthermore, the distance of 14.8 Å between S76_Rab1b_ and G108C_AnkX_ is in accordance with the length of the PC-based linker. However, the switch II loop of Rab1b including S76_Rab1b_ is not reaching into the catalytic pocket of AnkX, indicating that the structure may represent a postcatalytic complex. Commonly, the switch I region of GTPases in the inactive GDP-bound state illustrates high structural flexibility, whereas it is fully defined in the present structure (Rab1b: amino acids 30 to 43).

The crystal structure of AnkX_G108C_:PC:Rab1b:GDP revealed three interfaces for AnkX binding (Fig. 3B). An expected contact area is located between the catalytic AnkX FIC domain and the switch II of Rab1b, carrying the modified S76_Rab1b_. A second binding interface is observed between AnkX ARs 5 to 9 and the switch I of Rab1b, the latter being involved in protein interaction in many small GTPases (Fig. 3, C and D, and fig. S4). The third interface includes AnkX ARs 10 to 13 and the C terminus of Rab1b, which has not been described for any small GTPase so far (Fig. 3, C and D, and fig. S4).

To illustrate the structural changes that Rab1b undergoes upon binding to AnkX, the structure of Rab1b from the AnkX_G108C_:PC:Rab1b:GDP complex was superimposed and compared to the crystal structure of active Rab1b [PDB ID: 3NKV; root mean square deviation (RMSD), 0.45 Å; Fig. 3, E and F] (*10*). The active-state Rab1b thereby represents the structure of the small GTPase before binding to AnkX. Upon complex formation, the Rab1b G domain does not undergo structural rearrangements. Thus, upon AnkX binding, structural changes within Rab1b only take place in the switch regions, as is typical for small GTPases. In particular, the switch II region is experiencing major structural reorganization upon binding to AnkX.

To verify the relevance of the AnkX ARs, we assessed the activity of respective truncations thereof with a Western blot–based activity assay. Successive truncation of ARs 13 to 9 resulted in a stepwise decrease in enzymatic activity, thereby confirming the relevance of the C-terminal ARs observed in a previous analysis (Fig. 3G) (*15*). These results are in line with the temperature factors (B factors) of the ARs: ARs 13 and 12 feature high B factors, indicating structural mobility due to the weak interaction with Rab1b. In contrast, AR 11 and further ARs display low B factors, indicating rigidity due to interactions with the small GTPase (fig. S5).

In addition, selected alanine substitutions of AnkX amino acids from ARs 5 to 9 and ARs 10 to 13 affect phosphocholination of Rab1b in vitro: R637A_AnkX_ and Y631A_AnkX_ severely reduced the catalytic activity of AnkX, whereas R560A_AnkX_, N598A_AnkX_, and F740A_AnkX_ moderately influenced phosphocholination (Fig. 3H and fig. S4). R560_AnkX_ and N598_AnkX_ show polar contacts to S36_Rab1b_ and Y33_Rab1b_, respectively. R637_AnkX_ binds to D31_Rab1b_ via a salt bridge, and the aromatic ring of Y631_AnkX_ forms a hydrophobic contact with the aliphatic side chain of K153_Rab1b_ (Fig. 3, C and D, and fig. S4, A and B). However, F740_AnkX_ does not appear to form direct contacts in the complex crystal structure but is closely located to a hydrophobic pocket provided by the aliphatic side chains of K170_Rab1b_ and K171_Rab1b_ (fig. S4, C and D). The impact of the F740A_AnkX_ substitution on phosphocholination therefore suggests that this hydrophobic contact is relevant for Rab1b binding to AnkX. The amino acids for Rab1b binding and the sequence of the ARs are highly conserved among AnkX variants from different *Legionella* species, demonstrating that these enzymes likely share a similar target profile (fig. S6).

To validate the determined AnkX-Rab1b binding interfaces on Rab1b, we used an alanine substitution approach. Using mass spectrometry, the respective Rab1b Ala mutants were screened for phosphocholination by catalytic quantities of AnkX_WT_ (fig. S7). For selected Rab1b Ala variants, the catalytic efficiency (*k*_cat_/*K*_M_) was evaluated with a time-resolved tryptophane fluorescence–based assay (Fig. 3I) (*14*). Effectively, mutants with reduced catalytic efficiency could be observed in all areas of interaction, confirming the crystallographic results. AnkX interacts with switch I, switch II, and the C-terminal region of Rab1b. The effect of alanine substitution is most notable for residues of the switch II region that are in close proximity to the modified S76_Rab1b_, with catalytic efficiencies reduced up to 5% of wild-type Rab1b (Rab1b_WT_) (Fig. 3I).

For the C terminus of Rab1b, the alanine screening approach results in a rather moderate decrease in catalytic efficiencies (25 to 70% of Rab1b_WT_), indicating that the binding of the Rab1b C terminus by AnkX is mediated by cumulative interaction between several residues. These C-terminal interactions are highly atypical for small GTPases and suggest a unique recognition mode of Rab1b by AnkX.

The three initial residues of the Rab1b switch I (D30_Rab1b_, D31_Rab1b_, and T32_Rab1b_), which are commonly structurally ordered in the active and in the inactive state of the small GTPase, interact with ARs 8 and 9 (Fig. 3, A and F). In contrast, Rab1b residues T74_Rab1b_, S75_Rab1b_, Y77_Rab1b_, and R79_Rab1b_ of the switch II region, which are in close proximity to the modified S76_Rab1b_, a direct interaction partner on the side of AnkX, cannot be observed from the crystal structure. However, since the AnkX_G108C_:PC:Rab1b:GDP complex might be of postcatalytic nature, it is conceivable that these Rab1b residues interact with the AnkX FIC domain during the phosphocholination reaction. The residues of the C-terminal Rab1b binding interface that interact with ARs 10 to 13 are located within α4 (D141_Rab1b_), the loop between β6 and α5 (K153_Rab1b_ and N154_Rab1b_), and α5 (N157_Rab1b_, Q160_Rab1b_, and E168_Rab1b_).

### AnkX mediates phosphocholination by switch II displacement

Having verified the importance of the AnkX ARs for the binding of Rab1b, we aimed to investigate the catalytic role of the AnkX FIC domain for Rab1b switch II modification in further detail. To assess whether AnkX exhibits any structural changes upon binding of Rab1b, we superimposed the structure of AnkX_1–484_ bound to CDP-choline (PDB ID: 4BET) (*19*) to the AnkX_G108C_:PC:Rab1b:GDP ternary complex (RMSD, 0.91 Å). Upon binding of Rab1b, the AnkX FIC domain and the first four ARs do not show any fundamental structural changes.

**Fig. 4:**
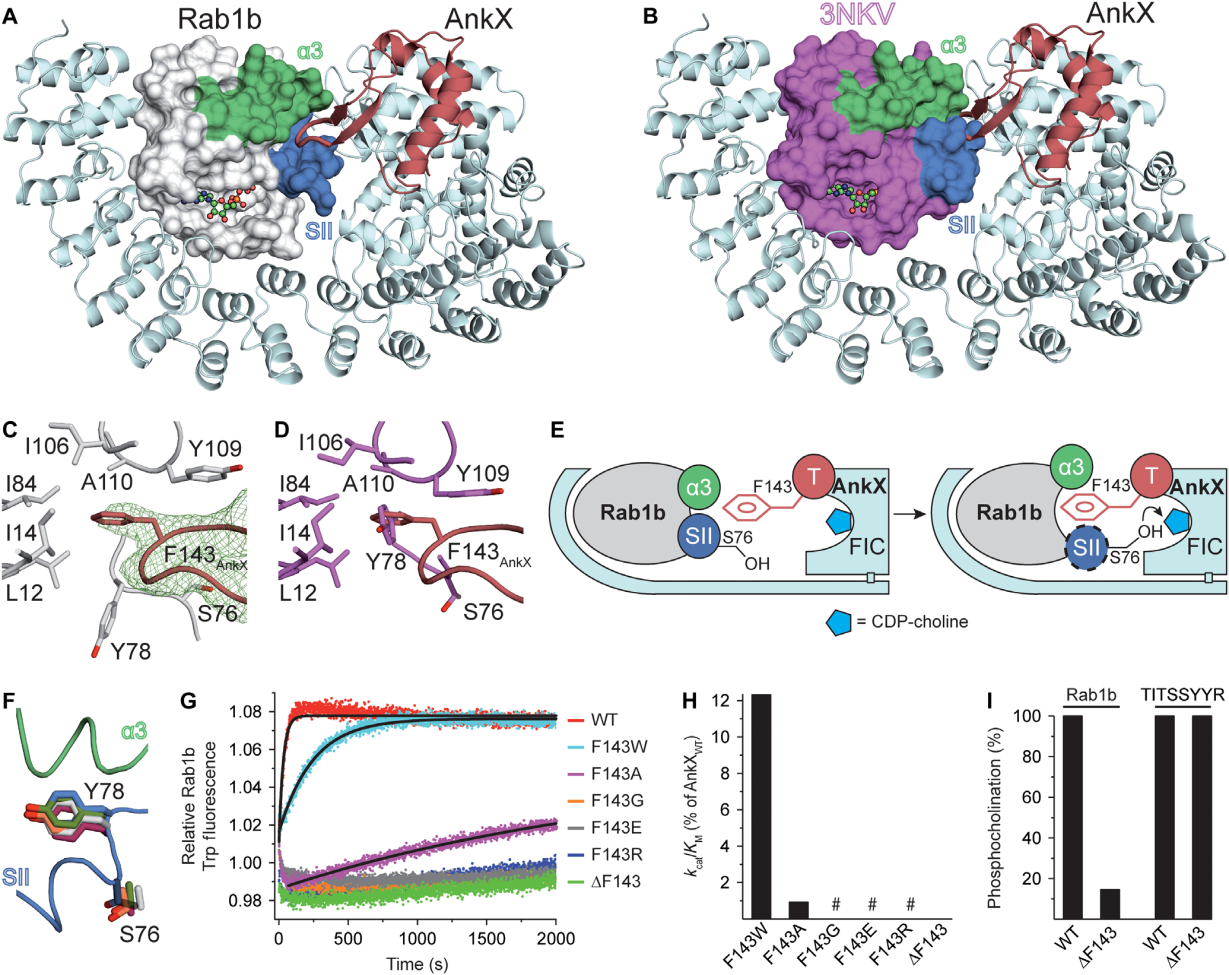
AnkX mediates phosphocholination by displacing switch II of Rab1b. (**A**) In the AnkX_G108C_:PC:Rab1b:GDP complex, the AnkX thorn inserts between switch II and α3 of Rab1b. AnkX (light blue) is illustrated as a cartoon model. Red, AnkX insertion domain bearing the AnkX thorn. Rab1b (gray) is illustrated as a surface model. Blue, Rab1b switch II; green, α3 of Rab1b. GDP is represented as sticks and spheres. (**B**) In active Rab1b (PDB ID: 3NKV) (*10*), the area between switch II and α3 is initially not accessible for the AnkX thorn. Active Rab1b (magenta) is illustrated as a surface model and was structurally superimposed with the AnkX_G108C_:PC:Rab1b:GDP complex. Labeling is the same as that in (A). (**C**) In the AnkX_G108C_:PC:Rab1b:GDP complex, F143_AnkX_ at the tip of the AnkX thorn (red) binds into a hydrophobic pocket of Rab1b (gray). (**D**) In active Rab1b (PDB ID: 3NKV) (*10*), the binding of F143_AnkX_ into the hydrophobic pocket of Rab1b is blocked by Y78_Rab1b_. (**E**) Scheme for the suggested role of the AnkX thorn during AnkX-mediated phosphocholination. By insertion of F143_AnkX_ at the tip of the AnkX thorn (T) into the hydrophobic pocket between switch II and α3 of Rab1b, AnkX locally unfolds the adjacent region of S76_Rab1b_ and thus enables S76_Rab1b_ to reach into the catalytic pocket of AnkX. (**F**) Structural superposition of active Rab1b (PDB ID: 3NKV; blue) (*10*) with crystal structures of GDP-bound Rab proteins: Rab2b:GDP (PDB ID: 2A5J; green), Rab3b:GDP (PDB ID: 3DZ8; gray), Rab4b:GDP (PDB ID: 2O52; orange), and Rab23:GDP (PDB ID: 1Z22; magenta). Residues at the position of Y78_Rab1b_ are highly conserved and thus severely restrict the structural mobility of related amino acids at the site of S76_Rab1b_ and related amino acids. Switch II and α3 of active Rab1b are depicted as cartoon model. (**G**) Time-resolved tryptophane fluorescence–based assay to monitor the catalytic activity of F143_AnkX_ mutants. Rab1b (5 μM) was incubated with CDP-choline (1 mM), and upon addition of catalytic amounts of F143_AnkX_ variants (250 nM), the tryptophane fluorescence of Rab1b was recorded. Fluorescence curves were fitted to a single exponential (black lines). (**H**) Catalytic efficiencies (*k*_cat_/*K*_M_ values) of the F143_AnkX_ mutants were calculated from the fluorescence curves of (G) [number sign (#) indicates not determined because of inactivity]. (**I**) Phosphocholination activity of the ΔF143_AnkX_ variant toward folded and unfolded AnkX substrates. Folded Rab1b protein (50 μM) or the unfolded octapeptide TITSSYYR (50 μM) was incubated with CDP-choline (10 mM) and either ΔF143_AnkX_ (5 μM) or WT_AnkX_ (5 μM) for 4 days. The degree of phosphocholination was assessed with mass spectrometry and plotted as bar chart.

Compared to other structurally characterized FIC enzymes, AnkX comprises an additional insert domain (amino acids 110 to 179) located in the conserved β-hairpin (*19*). Apart from three α helices, it contains a two-stranded β sheet that protrudes from the enzyme and adapts a rigid, thorn-like structure (Fig. 4A).

F143_AnkX_ is located at the tip of the thorn and binds into a hydrophobic pocket of Rab1b between switch II and α3 (Fig. 4, A and C). While this site is occupied by Y78_Rab1b_ in the structure of active Rab1b [PDB ID: 3NKV (*10*)] (Fig. 4B, D), F143_AnkX_ displaces it after AnkX binding (Fig. 4C).

Regarding the reasons and consequences of Y78_Rab1b_ displacement by F143_AnkX_ on to the enzymatic mechanism of PC modification at S76_Rab1b_, we speculated that S76_Rab1b_ is structurally fixed and cannot spontaneously reach into the catalytic center of AnkX. Previous observations showed that AnkX does not significantly discriminate between the active (GTP) and inactive (GDP) states of Rab1b and accepts peptides derived from the Rab1b switch II region as substrates (*14*, *23*). Since the switch II region is conformationally flexible in the inactive state, a stronger discrimination of the activity states by AnkX would have been expected if structural flexibility was the sole contributing factor. Therefore, it is possible that AnkX brings S76_Rab1b_ closer to its active site by locally displacing the adjacent region through F143_AnkX_-mediated displacement of Y78_Rab1b_ (Fig. 4E). Structural superpositions of several crystal structures of inactive, GDP-bound Rab proteins revealed that the orientation and positioning of Y78_Rab1b_ is highly conserved [PDB IDs: 2AJ5 (Rab2b), 2O52 (Rab4b), 1Z22 (Rab23), and 3DZ8 (Rab3b)] (*24*): Y78_Rab1b_ and corresponding residues in other Rab proteins are always aligned toward the same hydrophobic cavity (Fig. 4F). Hence, the positioning of Y78_Rab1b_ severely restricts the structural mobility of proximate S76_Rab1b_ due to its close vicinity. AnkX locally unfolds the GTPase switch II by F143_AnkX_-mediated displacement of Y78_Rab1b_. S76_Rab1b_ is then able to reach into the distant catalytic center of AnkX for phosphocholination.

To verify this hypothesis, the relevance of F143_AnkX_ for Rab1b phosphocholination was examined by assessing the consequences of amino acid substitutions on AnkX activity in a tryptophane fluorescence assay (Fig. 4G) (*14*). Only F143_AnkX_ variants with hydrophobic substitutions (tryptophane and alanine) were able to quantitatively phosphocholinate Rab1b. However, the catalytic efficiencies of these mutant proteins significantly decreased to 12% (F143W_AnkX_) and 1% (F143A_AnkX_) of AnkX_WT_ activity (Fig. 4H). The substitution of F143_AnkX_ with glycine or polar residues (glutamate or arginine) and the recombinant truncation of the AnkX thorn (amino acids 141 to 144, referred to as ΔF143_AnkX_) severely impaired enzyme activity to a degree where catalytic efficiencies could not be determined. Mass spectrometry data, on the other hand, suggest that F143G_AnkX_, F143E_AnkX_, and F143R_AnkX_ were able to partially phosphocholinate Rab1b within 8 hours, whereas no modified Rab1b was detected in the presence of ΔF143_AnkX_ (fig. S9). Furthermore, the substitution of Y78_Rab1b_ with alanine leads to increased phosphocholination of the small GTPase by AnkX_WT_ (fig. S7). These experiments confirm that the binding of F143_AnkX_ into the hydrophobic pocket and the associated displacement of Y78_Rab1b_ play a significant role for AnkX-mediated phosphocholination of Rab1b.

To further strengthen our hypothesis of switch II displacement, the phosphocholination activity of ΔF143_AnkX_ toward the native Rab1b protein was compared to the octapeptide TITSSYYR. This unfolded peptide is derived from the Rab1b switch II region and has previously been reported as a substrate for AnkX-mediated phosphocholination (*14*). After 4 days of incubation, TITSSYYR is quantitatively phosphocholinated by ΔF143_AnkX_, whereas the folded GTPase Rab1b is modified to less than 15% (Fig. 4I and fig. S9B). This demonstrates that F143_AnkX_ is not involved in the catalytic mechanism of AnkX but is required to unfold the Rab1b switch II, enhancing subsequent phosphocholination at S76_Rab1b_.

## DISCUSSION

Using a combination of organic synthesis and biochemistry, we successfully obtained the elusive complex structure of *Legionella* PC transferase AnkX and its mammalian target Rab1b. The crystal structure of this covalently linked enzyme-protein complex revealed further insight into the molecular mechanisms of substrate phosphocholination. AnkX binds to Rab1b using a previously unknown GTPase binding interface and inserts a thorn-like β sheet element into a structurally crucial intramolecular binding motif of Rab1b, located at the C-terminal end of switch II. This induces a functionally important displacement of the adjacent region. These findings explain why AnkX, in contrast to most other GTPase-interacting proteins, can accept the inactive and active Rab1b states alike.

Because of the lack of structural data, little is known about the recognition of protein substrates by FIC proteins. The complex between IbpA and AMPylated Cdc42 is the only example to date where the binding interface of a FIC protein and its protein substrate could be studied at the molecular level (*25*). Here, IbpA is binding and stabilizing the formerly flexible switch I region of Cdc42 via a β-hairpin next to the FIC motif and thereby positions the target tyrosine residue of Cdc42 precisely into the catalytic site of IbpA (fig. S10). In AnkX, the respective β-hairpin is buried by an additional domain containing a distinct thorn. Since accessibility of the β-hairpin is limited in AnkX, the binding mode of Rab1b remained elusive so far. Our structural data reveal that this thorn is a rigid but integral part for Rab1b recognition. By binding into a hydrophobic cavity between switch II and α3 of Rab1b, the AnkX thorn positions switch II into proximity of the β-hairpin. Only after this rearrangement, switch II is free to interact with the β-hairpin, allowing S76_Rab1b_ to penetrate the active site of AnkX. Whereas AnkX inserts the β-hairpin between switch II and helix α3 of Rab1b, the corresponding element in IbpA is positioned between switch I and switch II in Cdc42 (fig. S10). Consequently, the β-hairpin appears to have related functions in these enzymes since it displaces closely located structural elements in the GTPases, yet the mode of interaction is very divergent.

Interaction partners of Rab1b typically show a distinct preference for either the active (GTP) or inactive (GDP) state of the small GTPase. For example, the AMP-transferase domain of the *Legionella* effector DrrA prefers the GTP state of Rab1b by a factor of 300, while the GDP dissociation inhibitor (GDI) favors the GDP state by a factor of 100 to 1000 (*26*, *27*). AnkX does not substantially discriminate between the nucleotide states of Rab1b, with a twofold preference for GDP (*14*). Considering the necessary rearrangement of switch II for AnkX-mediated phosphocholination, a larger preference of AnkX for the GDP state, where the Rab1b switch II is intrinsically more flexible than in presence of GTP, would have been expected. However, structural superposition of several Rab proteins in the GDP state revealed that the orientation of S76_Rab1b_ and the adjacent Y78_Rab1b_ is highly conserved and very similar to the GTP state. Therefore, both the GTP form and the GDP form of switch II need to be locally unfolded to allow penetration of S76_Rab1b_ into the catalytic site of AnkX. Our structural and biochemical data demonstrate that F143_AnkX_ at the top of the AnkX thorn facilitates displacement of switch II by displacing Y78_Rab1b_ from its hydrophobic cavity and thereby primes S76_Rab1b_ for AnkX-mediated phosphocholination.

In addition, we found that AnkX requires a binding interface in Rab1b that has so far not been identified for small GTPase:protein interactions. The C-terminal ARs of AnkX make contacts to helices α4 and α5 of Rab1b. Consistently, recombinant truncations of these motifs result in severely decreased phosphocholination activity of AnkX (*15*). In general, interaction partners of small GTPases attach to switch I or switch II to ensure binding specificity and recognition of the G domain activity state [reviewed in (*6*)]. The C-terminal Rab1b region constituted by helices α4 and α5, however, is located at the opposite side of the GTPase, and since this part does not undergo structural changes upon nucleotide exchange, it was assumed to be not involved in contacts to GTPase interaction partners. In the case of AnkX, we presume that Rab1 binding is partially mediated by the interaction between the ARs and Rab1b helices α4 and α5 since the enzyme activity is decreased profoundly when omitting the amino acid region 689 to 800 in AnkX. Previous experiments demonstrated that AnkX modifies Rab1b even if the phosphocholination site at S76_Rab1b_ is blocked by the tightly bound eukaryotic Rab-recycling protein GDI. Upon phosphocholination, Rab1b is released from GDI (*14*, *20*). However, Rab1b helices α4 and α5 are not covered in the complex with GDI and available to AnkX binding. Therefore, AnkX may use this region for initial binding to the Rab1b:GDI complex and permit rapid phosphocholination after GDI dissociation.

It has been previously observed that the AnkX amino acid region 491 to 949 can attach to the plasma membrane and interacts with the lipids phosphatidyl-3-phosphate [PI(3)P] and phosphatidyl-4-phosphate [PI(4P)] (*28*). However, the truncation construct spanning amino acids 688 to 949 lost its membrane affinity. The membrane affinity of AnkX seems to depend on two separate events, i.e., partial Rab1b binding via AnkX 688 to 800 or 491 to 800 and PI(3)P/PI(4P) interactions within 491 to 949. With Rab interactions up until position 800 of AnkX, PI(3)P/PI (4P) binding activity is presumably located in the C-terminal domain from 801 to 949.

As previously demonstrated, AnkX is applicable for enzymatic labeling of arbitrary protein targets carrying recombinantly linked octapeptide recognition motifs in conjunction with synthetic functionalized CDP-choline derivatives (*23*). However, cytosolic in vivo labeling would compete with the naturally present Rab1 as prime target of AnkX, thereby precluding specific protein modification. The importance of the AnkX thorn for switch II displacement of Rab1b and phosphocholination could offer a solution to this problem: Recombinant deletion of the AnkX thorn tip abrogates Rab1 phosphocholination almost entirely while maintaining the ability to modify unfolded AnkX recognition motif peptides.

In summary, we have used a combination of chemical synthesis and biochemistry to investigate the elusive Rab1b-AnkX complex structure by x-ray crystallography. Unexpectedly, one of several distinct interaction surfaces between the ARs and Rab1 is the C-terminal part of the G domain. Furthermore, AnkX mediates displacement of switch II in Rab1b and thereby facilitates phosphocholination in the enzyme’s active center. This supports the previous observation that AnkX is able to modify switch II in the ordered (active) and disordered (inactive) conformation. We demonstrate that the utilization of functionalized nucleotide derivatives (e.g., CDP-choline chloroacetamides) is viable to obtain mechanistic insights into the action of FIC proteins (e.g., AnkX). Our approach represents a valuable concept for studying protein interactions between FIC enzymes and their targets.

## MATERIALS AND METHODS

### Molecular biology

The AnkX_1–800_ (referred to as AnkX)–encoding DNA, which previously had been amplified from *L. pneumophila* genomic DNA (*14*), was cloned into a modified pSF vector (Oxford Genetics) by sequence and ligation independent cloning [sequence and ligation independent cloning (SLIC)] using T4 DNA polymerase (New England Biolabs). This resulted in AnkX constructs with an N-terminal decahistidine (His_10_)–tag, followed by enhanced green fluorescent protein (eGFP) and the tobacco etch virus (TEV) protease cleavage site.

The Rab1b_3–174_ (referred to as Rab1b)–encoding DNA, which previously had been codon-optimized for expression in *Escherichia coli* by omitting rare amino acid codons (*29*), was cloned into a modified pMAL vector (New England Biolabs) by SLIC using T4 DNA polymerase (New England Biolabs). This resulted in Rab1b constructs with an N-terminal His_6_-tag, followed by maltose-binding protein (MBP), the TEV protease cleavage site, a Strep-tag, and the PreScission protease cleavage site. For our strategy to purify the AnkX_Cys_:PC:Rab1b complex, the Strep-tag of the Rab1b vector was replaced with a His_10_-tag by site-specific mutagenesis. All site-specific mutagenesis was performed with the Q5 Site-Directed Mutagenesis Kit (New England Biolabs).

### Protein expression and purification

For overexpression of proteins, *E. coli* BL21-CodonPlus (DE3) cells (for Rab1b constructs) or BL21-CodonPlus (DE3)-RIL cells (for AnkX constructs) were transformed with the respective plasmid and grown in 1 to 1.5 liters of lysogeny broth medium containing ampicillin (34 μg/ml) [and chloramphenicol (34 μg/ml) for BL21-CodonPlus (DE3)-RIL cells] at 37°C and 200 rpm in the Innova 44 incubator (New Brunswick). At OD_600_ (optical density at 600 nm) of 0.6 to 0.8, protein expression was induced by adding 0.5 mM isopropyl-β-d-thiogalactopyranoside, following overnight incubation at 20°C and 200 rpm. Cells were harvested by centrifugation at 5100 rpm and 20°C for 30 min (Sigma 8K centrifuge, Sigma Centrifuges). After resuspending and washing the pelleted bacteria in phosphate-buffered saline (PBS; 137 mM NaCl, 2.7 mM KCl, 10 mM NaH_2_PO_4_, and 2 mM KH_2_PO_4_), cells were centrifuged at 4000 rpm and 4°C for 30 min (Centrifuge 5810 R, Eppendorf). Afterward, cell pellets were resuspended in buffer A [50 mM tris, 500 mM NaCl, 5% (v/v) glycerol, and 2 mM β-ME (pH 8.0) for AnkX constructs or 50 mM Hepes, 500 mM NaCl, 1 mM MgCl_2_, 10 μM GDP, and 2 mM β-ME (pH 7.5) for Rab1b constructs]. To the resuspended cells, a spatula tip of deoxyribonuclease I (DNAse I) (Sigma-Aldrich) was added, and the cells were lysed with a French press (Constant Cell Disruption Systems) by applying a pressure of 2.2 kbar. Subsequently, 1 mM protease inhibitor phenylmethylsulfonyl fluoride (PMSF) was added, and the crude lysate was cleared from cell debris by centrifugation at 20,000 rpm and 4°C for 45 min (Avanti J-26 XP centrifuge, Beckman Coulter) using the JA-25.50 rotor.

For the isolation of AnkX and Rab1b proteins via their polyhistidine affinity tag, the cleared cell lysate was applied to metal chelate affinity chromatography using a 5-ml Nuvia IMAC column (Bio-Rad Laboratories) that was preloaded with NiSO_4_ and equilibrated with buffer A containing 25 mM imidazole. For all chromatography, the NGC medium-pressure liquid chromatography (LC) system (Bio-Rad Laboratories) was used. After adding 25 mM imidazole, the cleared lysate was loaded onto the column and washed with buffer A and 5% buffer B (buffer A containing 500 mM imidazole). Subsequently, a 95-ml gradient of buffer B (5 to 100%) was applied, where target proteins eluted at 20 to 40%. Protein-containing fractions were identified by SDS-PAGE and pooled. Afterward, the proteins were dialyzed against 3 liters of dialysis buffer [20 mM tris, 300 mM NaCl, 5% (v/v) glycerol, and 2 mM β-ME (pH 8.0) for AnkX constructs or 20 mM Hepes, 100 mM NaCl, 1 mM MgCl_2_, 10 μM GDP, and 2 mM β-ME (pH 7.5) for Rab1b constructs] overnight at 4°C. During dialysis, the target proteins were incubated with 25 μg of TEV (AnkX constructs and the Rab1b construct for AnkX_Cys_:PC:Rab1b complex formation) or PreScission protease (all other Rab1b constructs) per milligram of protein to cleave the polyhistidine affinity tag and the eGFP or MBP solubility tag. To purify the proteins of interest from the protease and the cleaved tags, reverse metal chelate affinity chromatography was applied, either collecting the target protein in the flow through (Rab1b constructs) or eluting it with buffer A containing 20 mM imidazole (AnkX constructs). The His_10_-PreScission-Rab1b construct for AnkX_Cys_:PC:Rab1b complex formation was purified from the cleaved His_6_-MBP-tag by reverse amylose affinity chromatography using a 5-ml MBPTrap HP column (GE Healthcare Life Sciences).

To separate monomeric from oligomeric species, proteins were further purified by size exclusion chromatography using a 16/600 Superdex 75 pg column (GE Healthcare Life Sciences). The column was equilibrated with gel filtration buffer [20 mM tris, 300 mM NaCl, 5% (v/v) glycerol, and 1 mM β-ME (pH 8.0) for AnkX constructs or 20 mM Hepes, 50 mM NaCl, 1 mM MgCl_2_, 10 μM GDP, and 1 mM β-ME (pH 7.5) for Rab1b constructs]. Fractions containing the protein of interest were identified by SDS-PAGE and concentrated to 10 to 30 mg/ml using Amicon Ultra 15-ml centrifugal filters (Merck Millipore).

### Protein crystallization and structure determination

The AnkX_G108C_:PC:Rab1b:GDP complex was applied in sitting-drop crystallization by vapor diffusion at 20°C. Sufficiently diffracting crystals were obtained after 1 to 2 weeks with 3.5 M sodium formate (pH 6.5) as precipitant in a drop containing 0.5 μl of protein (25 mg/ml) and 0.2 μl of reservoir solution. Crystals were soaked with cryo-protectant (1:1 reservoir and glycerol) and flash-frozen with liquid nitrogen. Datasets were collected at 3.2-Å resolution at beamline X06SA (Swiss Light Source, Paul Scherrer Institute, Villigen, Switzerland; table S1). Diffraction intensities were evaluated by X-ray detector software (XDS) (*30*). Cell parameters and the monoclinic space group C2 indicated a heterodimeric assembly in the asymmetric unit at a solvent content of 70%. The structures of AnkX_1–484_ [PDB ID: 4BES (*19*)] and Rab1b:AMP [PDB ID: 3NKV (*10*)] were used for molecular replacement by Patterson search algorithms with Phenix (*31*). Further model building was performed using Coot (*32*) with restrained refinements by REFMAC5 in between (*33*). The structure was finalized by translation/liberation/screw and restrained refinements until suitable values for *R*_work_/*R*_free_ and all geometric parameters were achieved (table S1). The crystal structure was deposited in the Research Collaboratory for Structural Bioinformatics (RCSB) PDB under accession code 6SKU.

### Preparation of *E. coli* lysates and protein purification with magnetic beads

For the preparation of *E. coli* lysates and protein purification with magnetic beads in a small scale, overexpression of proteins was performed as described above but was restricted to 20 ml of bacterial culture. After overnight expression of proteins, 2 ml of bacterial culture was harvested by centrifugation at 7000 rpm for 2 min (Centrifuge 5424, Eppendorf). After resuspending and washing the pelleted bacteria in PBS, cells were centrifuged at 7000 rpm for 2 min (Centrifuge 5424, Eppendorf). To lyse the cells, the cell pellet was resuspended in 20 μl of xTractor Buffer (Clontech Laboratories) per milligram of pellet. PMSF (1 mM), a spatula tip of DNAse I, and a spatula tip of lysozyme (Carl Roth) were added to the resuspended cells, and the cells were incubated at 4°C for 1 hour under continuous rotating in an Intelli-Mixer (NeoLab). Thereafter, the crude lysate was cleared from cell debris by centrifugation at 13,000 rpm and 4°C for 30 min (Centrifuge 5424 R, Eppendorf). The cleared lysate containing the overexpressed target protein was either directly used for further experiments (AnkX AR truncations) or subjected to magnetic bead–based affinity purification via the Strep-tag (Rab1b Ala mutants).

For affinity purification of Rab1b, constructs via the Strep-tag MagStrep “type3” XT beads (IBA Lifesciences) were used. Thirty microliters of magnetic bead suspension containing 1.5 μl of magnetic beads were equilibrated twice with 300 μl of Rab1b gel filtration buffer [20 mM Hepes, 50 mM NaCl, 1 mM MgCl_2_, 10 μM GDP, and 1 mM β-ME (pH 7.5)] using a magnetic separator. Subsequently, the magnetic beads were mixed with the cleared lysate and incubated at 4°C for 1 hour under continuous rotating in the Intelli-Mixer (NeoLab). Thereafter, the beads were washed three times with 900 μl of Rab1b gel filtration buffer using a magnetic separator. To elute the target protein, the beads were incubated with 50 μl of Rab1b gel filtration buffer containing 5 μg of PreScission protease overnight at 4°C under continuous rotating in the Intelli-Mixer (NeoLab). With a magnetic separator, the supernatant containing the target protein was separated from the magnetic beads and collected.

### Analytical AnkX_Cys_:PC:Rab1b complex formation

#### Direct approach

To assess the ability of recombinant AnkX versions with strategically placed cysteines (AnkX_Cys_) to form the covalent AnkX_Cys_:PC:Rab1b complex in the direct approach, 50 μM AnkX_Cys_ was incubated with 1 mM thiol-reactive CDP-choline analog at 20°C overnight in adduct buffer [20 mM tris, 300 mM NaCl, 5% (v/v) glycerol, and 1 mM β-ME (pH 8.0)]. For initial experiments, the binary adduct between AnkX_Cys_ and CDP-choline derivative was not purified but directly incubated with 100 μM Rab1b overnight at 20°C. Subsequently, formation of the AnkX_Cys_:PC:Rab1b complex was verified by SDS-PAGE gel shift assay.

During further optimization of the direct approach, the binary adduct between AnkX_Cys_ and CDP-choline analog was purified from excess nucleotide by exchanging the buffer three times with adduct buffer using Amicon Ultra 0.5-ml centrifugal filters (Merck Millipore) before the addition of Rab1b. With this additional purification step, unspecific AnkX_Cys_:PC:Rab1b complexes could be prevented.

To optimize the binary adduct formation, 50 μM AnkX_D265C_ and 1 mM CDP-choline analogs were incubated at different temperatures (20°, 25°, 30°, and 37°C) and for different time periods (12, 24, 48, and 72 hours). After removal of excess nucleotide, 50 μM binary conjugate was incubated with 100 μM Rab1b overnight at 20°C, and AnkX_D265C_:PC:Rab1b complex formation was evaluated by SDS-PAGE gel shift assay.

To optimize the formation of the AnkX_D265C_:PC:Rab1b complex, the binary conjugate between AnkX_D265C_ (50 μM) and CDP-choline derivative (1 mM) was produced at 37°C for 48 hours and purified from excess nucleotide. Thereafter, 50 μM binary adduct was incubated with 100 μM Rab1b at different temperatures (20°, 25°, 30°, and 37°C) and, for different time periods (2, 4, 6, and 24 hours) before the yield of AnkX_D265C_:PC:Rab1b complex, was quantified by SDS-PAGE gel shift assay.

#### Indirect approach

To test the capability of AnkX_Cys_ variants to form the AnkX_Cys_:PC:Rab1b complex in the indirect approach, Rab1b (50 μM) was quantitatively modified with thiol-reactive CDP-choline analogs (1 mM) by catalytic amounts of AnkX_WT_ (100 nM) at 20°C overnight in Rab1b gel filtration buffer [20 mM Hepes, 50 mM NaCl, 1 mM MgCl_2_, 10 μM GDP, and 1 mM β-ME (pH 7.5)]. Excess of nucleotide was purified using illustra NAP-5 columns (GE Healthcare Life Sciences). Afterward, 50 μM Rab1b-PC was incubated with 200 μM AnkX_Cys_ at 20°C overnight, and AnkX_Cys_:PC:Rab1b complex formation was assessed by SDS-PAGE gel shift assay.

For further experiments with the indirect approach including preparative generation of the AnkX_G108C_:PC:Rab1b complex, the cysteines C48, C84, and C172 of AnkX_G108C_ were mutated to serines to prevent unspecific AnkX_G108C_:PC:Rab1b complexes. To optimize the formation of the AnkX_G108C_:PC:Rab1b complex, 50 μM Rab1b-PC and 200 μM AnkX_G108C_ were incubated at 20°C for different time periods (1, 2, 4, 8, 24, and 48 hours), and the yield of AnkX_G108C_:PC:Rab1b complex was quantified by SDS-PAGE gel shift assay.

### SDS-PAGE gel shift assay

Samples from the direct (1 μg of AnkX) or indirect (4 μg of AnkX) approach were boiled in Laemmli buffer [50 mM tris, 10% (v/v) glycerol, 2% SDS, 200 mM β-ME, and 0.01% Bromophenol blue (pH 6.8)] for 5 min at 95°C and applied to SDS-PAGE using 12% acrylamide gels. To visualize the shift of the AnkX_Cys_:PC:Rab1b complex, gels were stained with Coomassie Brilliant Blue. To determine the yield of AnkX_Cys_:PC:Rab1b complex, the intensity of Coomassie-stained gel bands was quantified with the Image Studio Lite software (LI-COR Biosciences).

### Preparative AnkX_Cys_:PC:Rab1b complex formation

#### Direct approach (AnkX_D265C_:PC:Rab1b:GDP)

To form the AnkX_D265C_:PC:Rab1b:GDP complex with the direct approach, AnkX_D265C_ and His_10_-PreScission-Rab1b were expressed and purified as described above. AnkX_D265C_ and C3-Cl were incubated at a molar ratio of 1:1.2 at 30°C for 72 hours. Excess of nucleotide was separated by exchanging the buffer three times with adduct buffer [20 mM tris, 300 mM NaCl, 5% (v/v) glycerol, and 1 mM β-ME (pH 8.0)] using Amicon Ultra 15-ml centrifugal filters (Merck Millipore). The purified binary conjugate was incubated with His_10_-PreScission-Rab1b at a molar ratio of 1:1 overnight at 20°C.

To separate unreacted binary adduct, the ternary complex was subjected to metal chelate affinity chromatography using a 5-ml Nuvia IMAC column (Bio-Rad Laboratories) that was equilibrated in Rab1b buffer A [50 mM Hepes, 500 mM NaCl, 1 mM MgCl_2_, 10 μM GDP, and 2 mM β-ME at (pH 7.5)]. After washing with buffer A and 10% buffer B (buffer A containing 500 mM imidazole), a 90-ml gradient of buffer B (10 to 100%) was applied, where the ternary complex eluted at around 40%. The ternary complex was then dialyzed against 3 liters of dialysis buffer [20 mM Hepes, 100 mM NaCl, 1 mM MgCl_2_, 10 μM GDP, and 2 mM β-ME (pH 7.5)] overnight at 4°C and incubated with 25 μg of PreScission protease per milligram of protein to cleave the His_10_-tag.

The AnkX_D265C_:PC:Rab1b:GDP complex was purified from proteases, the cleaved His_10_-tag and unreacted Rab1b by size exclusion chromatography using a 16/600 Superdex 200 pg column (GE Healthcare Life Sciences). The column was equilibrated with complex buffer [20 mM Hepes, 50 mM NaCl, 1 mM MgCl_2_, 10 μM GDP, and 1 mM DTT (pH 7.5)]. Fractions containing the AnkX_D265C_:PC:Rab1b:GDP complex were identified by SDS-PAGE and concentrated to 12.5 mg/ml using Amicon Ultra 15-ml centrifugal filters (Merck Millipore).

#### Indirect approach (AnkX_G108C_:PC:Rab1b:GDP)

To form the AnkX_G108C_:PC:Rab1b:GDP complex with the indirect approach, AnkX_G108C_ (cysteines C48, C84, and C172 of AnkX_G108C_ mutated to serines) and His_10_-PreScission-Rab1b were expressed and purified as described above. His_10_-PreScission-Rab1b was modified with 1.2-fold excess of C3-Cl by AnkX_WT_ at a molar ratio of 1:500 overnight at 20°C. Excess nucleotide was separated by exchanging the buffer three times with Rab1b gel filtration buffer [20 mM Hepes, 50 mM NaCl, 1 mM MgCl_2_, 10 μM GDP, and 1 mM β-ME (pH 7.5)] using Amicon Ultra 15-ml centrifugal filters (Merck Millipore). The phosphocholinated His_10_-PreScission-Rab1b and AnkX_G108C_ were incubated at a molar ratio of 1:1 at 20°C for 72 hours. Purification of the ternary complex was performed as described for the indirect approach.

### Analytical size exclusion chromatography AnkX_Cys_:PC:Rab1b complexes

To assess the quality of protein preparations, samples of purified proteins (20 ng for Rab1b, 90 ng for AnkX, and 70 ng for the AnkX_Cys_:PC:Rab1b complex) were subjected to analytical size exclusion chromatography using a 10/300 Superdex 200 pg column (GE Healthcare Life Sciences). The column was calibrated with the Bio-Rad gel filtration standard (Bio-Rad Laboratories) to allow determination of the apparent molecular weight of target proteins. All size exclusion runs were performed on a high-pressure LC (HPLC) system (Shimadzu).

### Western blot–based activity assay of AnkX AR truncations

*E. coli* lysates overexpressing AnkX AR truncations were prepared as described above. Rab1b (5 μM) was phosphocholinated with CDP-choline (1 mM) by total cell lysate (0.5 mg/ml) for 2 hours at room temperature. Subsequently, samples were boiled in Laemmli buffer and subjected to Western blot analysis to assess the degree of Rab1b phosphocholination. The Western blots were quantitatively evaluated by densitometry using the Image Studio Lite software (LI-COR Biosciences), whereas the signal of phosphocholinated Rab1b was normalized to the signal of His_6_-AnkX AR truncations.

### Western blot analysis of phosphocholinated proteins

Phosphocholinated samples containing 500 ng of Rab1b were applied to SDS-PAGE, and proteins were transferred onto polyvinylidene fluoride (PVDF) membranes using Whatman paper, which was soaked in transfer buffer (48 mM tris, 39 mM glycine, 1.3 mM SDS, and 20% methanol). For the blotting procedure, the V20-SDP semidry blotter (SCIE-PLAS) was used, and a current of 0.7 mA/cm^2^ was applied for 2 hours. After blotting, the PVDF membrane was blocked with Roti-Block (Carl Roth) in tris-buffered saline containing 0.1% Tween 20 (TBS-T) for 1 hour. Subsequently, the α-PC antibody (clone TEPC 15, Sigma-Aldrich) was added into the blocking solution in a 1:1000 dilution and incubated overnight at 4°C with continuous shaking. Afterward, the membrane was washed three times with TBS-T for 10 min and then incubated with a secondary goat anti-mouse antibody-peroxidase conjugate (Thermo Fisher Scientific) in TBS-T for 45 min with continuous shaking. To remove unbound antibody, the membrane was washed in TBS-T three times for 10 min with continuous shaking. After imaging phosphocholinated Rab1b proteins, the membrane was stripped using ROTI Free Stripping Buffer (Roth) for 20 min at 60°C with continuous shaking. After reblocking the membrane, the α-His antibody (1:1000) was added into the blocking solution in a 1:1000 dilution and incubated overnight at 4°C with continuous shaking. Washing steps and incubation of secondary antibody was performed as described above. The peroxidase signal was detected with the SuperSignal West Dura (Thermo Fisher Scientific), and chemiluminescence was imaged using the Intas ECL ChemoCam (Intas Science Imaging). Statistical significance was evaluated using the paired Student’s *t* test.

### Mass spectrometry–based activity assay of Rab1b Ala mutants

Rab1b Ala mutants (1 μM) were modified with CDP-choline (50 μM) by catalytic amounts of AnkX (3.75 nM) for 1 hour at room temperature. Subsequently, samples were quenched by dilution in acetonitrile at a ratio of 1:1 and subjected to mass spectrometry. The degree of Rab1b phosphocholination was determined by quantifying the area of the deconvoluted mass peaks of modified and unmodified Rab1b using the software OriginPro (OriginLab) and normalized to Rab1b_WT_.

### LC/electrospray ionization mass spectrometry of phosphocholinated proteins

To verify the degree of phosphocholination, samples containing 100 ng of Rab1b were run over a 5-μm Jupiter C4 300 Å LC column (Phenomenex) using the 1260 Infinity LC system (Agilent Technologies) and then subjected to mass spectrometry with the 6100 Quadrupole LC/MS System (Agilent Technologies). The resulting ion spectra were deconvoluted using the Magic Transformer software (*34*) .

### Fluorescence spectrometry

For kinetic investigations of AnkX activity, the addition of the PC group on Rab1b was monitored via intrinsic tryptophane fluorescence of Rab1b using either the fluorescence spectrometer FluoroMax-4 (Horiba Jobin Yvon; λexc, 297 nm; λem, 340 nm; excitation slit, 1 nm; emission slit, 5 nm) or the plate reader Tecan Spark (Tecan; λexc, 297 nm; λem, 340 nm; excitation slit, 5 nm; emission slit, 5 nm). Measurements were conducted at 25°C in 20 mM Hepes, 50 mM NaCl, 1 mM MgCl_2_, 10 μM GDP, and 1 mM β-ME (pH 7.5). Rab1b (5 μM) and CDP-choline (1 mM) were provided in the cuvette (Hellma) or a 96-well plate (reference 655076, Greiner Bio-One), and phosphocholination was started by the addition of AnkX (50, 100, or 250 nM).

### Determination of catalytic efficiencies

For the determination of catalytic efficiencies (*k*_cat_/*K*_M_) of phosphocholination reactions measured by fluorescence spectrometry, reaction curves were fitted to a single exponential according to Eq. 1 using the software OriginPro (OriginLab). The resulting observed rate constant (*k*_obs_) was divided by the applied AnkX concentration (100 or 250 nM), yielding *k*_cat_/*K*_M_

$$F\;(t)=F_{0}+F_{A}\cdot e^{-k\text{obs}/t}$$ (1)

where *F*(*t*) is the fluorescence intensity, *F*_0_ is the minimum fluorescence intensity, *F*_A_ is the total fluorescence amplitude (i.e., *F*_max_ − *F*_0_, with *F*_max_ as the maximum fluorescence intensity), and *k*_obs_ is the observed rate constant. Statistical significance was evaluated using the paired Student’s *t* test.

### Chemical synthesis of CDP-choline derivatives

#### General methods

Chemicals were obtained from Sigma-Aldrich and Carbosynth and were used without further purification except when noted. Solvents were used as received or passed over a drying column [*N*,*N*′-dimethylformamide (DMF) and tetrahydrofuran]. Nuclear magnetic resonance (NMR) spectra were recorded on either a 400-MHz Varian Mercury-Oxford or a Bruker AVANCE (600 MHz) spectrometer. ^1^H chemical shifts are reported in δ values relative to tetramethylsilane and referenced to the residual solvent peak [CDCl_3_, δ_H_ = 7.26 parts per million (ppm) and δ_C_ = 77.16 ppm; D_2_O, δ_H_ = 4.79 ppm]. Coupling constants are reported in hertz. HPLC measurements were performed using an Agilent 1100 Series instrument. High-resolution mass spectra were recorded on an Agilent 6230 Series time-of-flight mass spectrometer coupled to an Agilent 1290 Infinity II LC system [HPLC column: Agilent, EclipsePlus C18, 50 mm by 2.1 mm, particle size (1.8 μm); ionization method: electrospray ionization (ESI)]. HPLC purification was performed on an Agilent 1260 Infinity instrument (HPLC column: Luna 5u C18, 250 mm by 21.2 mm).

#### Experimental procedures and spectral data

**3-Bromopropane choline (bromide salt) (S1).** *N*,*N*-dimethyl-ethanolamine (1.1 g, 1.3 ml, 12.6 mmol) was added to 1,3-dibromopropane (39.6 g, 20 ml, 196 mmol, 15.5 equivalent) under an argon atmosphere and allowed to stir over night at room temperature. After full conversion of *N*,*N*-dimethyl-ethanolamine (by ^1^H NMR), ice-cold diethyl ether (20 ml) was added, and the formed precipitate was filtered, washed with ice-cold diethyl ether (3 × 5 ml), and dried to yield the title compound as white solid (3.3 g, 11.3 mmol, 91%). ^1^H NMR (400 MHz, D_2_O): δ 4.09 to 4.04 (m, 2H), 3.62 to 3.50 (m, 6H), 3.19 (s, 6H), and 2.40 (m, 2H); ^13^C NMR (100 MHz, D_2_O): δ 65.15 (t, *J* = 3.0 Hz), 63.97 (t, *J* = 3.0 Hz), 55.32, 51.60 (t, *J* = 3.7 Hz), 29.00, and 25.11; ESI-high resolution mass spectrometry (HRMS) calculated mass [M]^+^ = 210.0488 and observed mass [M]^+^ = 210.0504.

**4-Bromobutane choline (bromide salt) (S2)** was synthesized accordingly from 1,4 dibromobutante and isolated as a slight brown solid (2.4 g, 10,7 mmol, 85%). ^1^H NMR (400 MHz, D_2_O): δ 3.99 to 3.95 (m, 2H), 3.49 to 3.42 (m, 4H), 3.37 to 3.30 (m, 2H), 3.08 (s, 6H), and 1.93 to 1.81 (m, 4H); ^13^C NMR (100 MHz, D_2_O): δ 64.92 (t, *J* = 3.0 Hz), 64.26 (t, *J* = 3.0 Hz), 55.33, 51.42 (t, *J* = 3.7 Hz), 33.20, 28.50, and 20.91; ESI-HRMS calculated mass [M]^+^ = 224.0645 and observed mass [M]^+^ = 224.0689.

**3-Azidopropane choline (bromide salt) (S3). S1** (2.0 g, 6.9 mmol) was dissolved in DMF (20 ml) and NaN_3_ (0.9 g, 13.7 mmol, 2 equivalent) was added to the stirring solution. The reaction mixture was heated to 70°C and stirred overnight. The formed suspension was allowed to cool to room temperature and filtered. The filtrate was evaporated under reduced pressure, and residual DMF was removed by coevaporation with toluene (3 × 5 ml). Final drying under high vacuum yielded the title compound as yellowish solid (1.5 g, 5.9 mmol, 86%). ^1^H NMR (400 MHz, D_2_O): δ 4.09 to 4.04 (m, 2H), 3.55 to 3.48 (m, 6H), 3.18 (s, 6H), and 2.18 to 2.03 (m, 2H); ^13^C NMR (100 MHz, D_2_O): δ 65.07 (t, *J* = 2.9 Hz), 62.78 (t, *J* = 3.1 Hz), 55.30, 51.51 (t, *J* = 3.9 Hz), 47.82, and 21.96.; ESI-HRMS calculated mass [M]^+^ = 173.1397 and observed mass [M]^+^ = 173.1412.

**4-Azidobutane choline (bromide salt) (S4)** was synthesized accordingly from 4-bromobutane choline (**S2**) and isolated as a yellowish oil (1,74 g, 9,3 mmol, 87%). ^1^H NMR (400 MHz, D_2_O): δ 3.98 to 3.94 (m, 2H), 3.45 to 3.41 (m, 2H), 3.36 to 3.32 (m, 4H), 3.08 (s, 6H), 1.85 to 1.76 (m, 2H), and 1.61 to 1.54 (m, 2H); ^13^C NMR (100 MHz, D_2_O): δ 64.89 (t, *J* = 2.9 Hz), 64.64 (t, *J* = 2.9 Hz), 55.31, 51.35 (t, *J* = 3.7 Hz), 50.33, 24.91, and 19.57; ESI-HRMS calculated mass [M]^+^ = 187.1553 and observed mass [M]^+^ = 187.1548.

**3-Azidopropane PC (S5).** POCl_3_ (472 mg, 3 mmol, 1.3 equivalent) was dissolved in dry acetonitrile (8 ml) under an argon atmosphere, and the solution was cooled with an ice bath before 2,6-lutidine (1 g, 9.5 mmol, 4 equivalent) was added to the stirred solution. **S3** (600 mg, 2.4 mmol) was dissolved in dry acetonitrile (2 ml) and added dropwise to the cooled reaction mixture. The reaction was stirred for 30 min on ice and subsequently quenched with ice-cold water. The acetonitrile was removed under reduced pressure, and the residual aqueous solution was basified (pH 13 to 14) with Ca(OH)_2_. The resulting slurry was filtered and evaporated to dryness yielding the title compound as a colorless oil (369 mg, 1.46 mmol, 61%). ^1^H NMR (600 MHz, D_2_O): δ 4.30 (m, 2H), 3.71 to 3.61 (m, 2H), 3.51 to 3.44 (m, 4H), 3.17 (s, 6H), and 2.12 to 2.05 (m, 2H); ^13^C NMR (151 MHz, D_2_O): δ 63.66, 62.96, 59.00 (d, *J* = 4.7 Hz), 51.52, 47.78, and 21.93; ^31^P NMR (162 MHz, D_2_O): δ −0.64; ESI-HRMS calculated mass [M]^+^ = 253.1060 and observed mass [M]^+^ = 253.1083.

**4-Azidobutane PC (S6)** was synthesized accordingly from 4-azidobutane choline (**S4**) and isolated as a yellowish oil (611 mg, 2,3 mmol, 68%). ^1^H NMR (400 MHz, D_2_O): δ 4,07 to 3.98 (m, 2H), 3.51 to 3.47 (m, 2H), 3.37 to 3.30 (m, 4H), 3.08 (s, 6H), 1.84 to 1.76 (m, 2H), and 1.62 to 1.54 (m, 2H); ^13^C NMR (100 MHz, D_2_O): δ 65.03 (t, *J* = 2.9 Hz), 64.22 (t, *J* = 2.9 Hz), 57.76, 51.31 (t, *J* = 3.7 Hz), 50.41, 24.98, and 19.57; ^31^P NMR (162 MHz, D_2_O): δ 3.26; ESI-HRMS calculated mass [M]^+^ = 267.1217 and observed mass [M]^+^ = 267.1289.

**3-Azidopropane CDP-choline (S7). S5** (117 mg, 464 μmol) and cytidin-5′-phosphomorpholidate 4-morpholine-*N*,*N*′-dicyclohexylcarboxamidium salt (635 mg, 928 μmol, 2 equivalent) were suspended in benzene (6 ml), frozen in liquid nitrogen, and lyophilized to remove traces of water. The dried residue was dissolved in dry pyridine (6 ml) under an argon atmosphere. 1*H*-tetrazole (64 mg, 928 μmol, 2 equivalent), evaporated from acetonitrile (2.1 ml, 0.45 M), was dissolved in dry pyridine (2 ml) under an argon atmosphere and subsequently dropped to the stirring reaction mixture. The reaction was heated to 60°C and stirred under argon overnight. The solvent was evaporated under reduced pressure, and the residue was purified by C18 HPLC to yield CDP-choline analog **S7** (133 mg, 239 μmol, 52%). ^1^H NMR (400 MHz, D_2_O): δ 8.22 (d, *J* = 8.0 Hz, 1H), 6.32 (d, *J* = 8.0 Hz, 1H), 5.95 (d, *J* = 3.4 Hz, 1H), 4.41 (m, 2H), 4.38 to 4.23 (m, 4H), 4.20 (m, 1H), 3.76 to 3.65 (m, 2H), 3.60 to 3.42 (m, 4H), 3.21 (s, 6H), and 2.16 to 2.05 (m, 2H); ^13^C NMR (100 MHz, D_2_O): δ 159.08, 148.41, 144.09, 95.20, 89.56, 83.21 (d, *J* = 9.1 Hz), 74.29, 69.01, 64.39, 63.03, 62.69, 59.63 (d, *J* = 5.1 Hz), 51.49, 47.79, and 21.95; ^31^P NMR (162 MHz, D_2_O): δ −11.38 (d, *J* = 21.2 Hz) and −12.18 (d, *J* = 21.2 Hz); ESI-HRMS calculated mass [M]^+^ = 558.1473 and observed mass [M]^+^ = 558.1467.

**4-Azidobutane CDP-choline (S8)** was synthesized accordingly from 4-azidobutane PC (**S6**) and isolated as a white solid (230 mg, 402 μmol, 49%). ^1^H NMR (400 MHz, D_2_O): δ 8.15 (d, *J* = 8.0 Hz, 1H), 6.22 (d, *J* = 8.0 Hz, 1H), 5.85 (d, *J* = 3.4 Hz, 1H), 4.31 (m, 2H), 4.28 to 4.20 (m, 4H), 4.14 (m, 1H), 3.70 to 3.53 (m, 4H), 3.47 to 3.28 (m, 2H), 3.10 (s, 6H), 1.85 to 1.77 (m, 2H), and 1.61 to 1.54 (m, 2H); ^13^C NMR (100 MHz, D_2_O): δ 163.43, 148.41, 144.11, 95.21, 89.57, 83.25 (d, *J* = 9.1 Hz), 74.30, 69.01, 66.82, 65.88, 63.55, 59.64, 50.37 (d, *J* = 5.1 Hz), 46.12, 40.52, and 19.56; ^31^P NMR (162 MHz, D_2_O): δ −11.40 (d, *J* = 13.2 Hz) and −12.17 (d, *J* = 13.2 Hz); ESI-HRMS calculated mass [M]^+^ = 572.1630 and observed mass [M]^+^ = 572.1701.

**3-Aminopropane CDP-choline (S9).** Pd/C (20 mg) was suspended in methanol (4 ml) under an argon atmosphere. Subsequently **S7** (130 mg, 233 μmol) was dissolved in methanol/H_2_O (2 ml, 1:1 v/v) and added to the stirring reaction mixture. The reaction was flushed with hydrogen and stirred for 4 hours at room temperature. After completion [by thin-layer chromatography (TLC) analysis], the suspension was filtered over celite in a sintered funnel and was washed with several portions of methanol. The solvent was removed under reduced pressure to yield the title compound as colorless solid (68 mg, 124 μmol, 54%). ^1^H NMR (600 MHz, D_2_O): δ 8.16 (d, *J* = 8.0 Hz, 1H), 6.29 (d, *J* = 8.0 Hz, 1H), 5.91 (d, *J* = 3.8 Hz, 1H), 4.40 (m, 2H), 4.31 (m, 4H), 4.18 (m, 1H), 3.76 to 3.71 (m, 2H), 3.58 to 3.52 (m, 2H), 3.21 (s, 6H), 3.10 (t, *J* = 7.5 Hz, 2H), and 2.28 to 2.20 (m, 2H); ^13^C NMR (151 MHz, D_2_O): δ 159.05, 148.31, 144.01, 95.18, 89.68, 83.09 (d, *J* = 9.0 Hz), 74.22, 69.03, 64.51, 63.35, 61.36, 59.71 (d, *J* = 5.1 Hz), 51.93, 36.22, and 20.67; ^31^P NMR (162 MHz, D_2_O): δ −11.27 (d, *J* = 20.5 Hz) and −12.14 (d, *J* = 20.5 Hz); ESI-HRMS calculated mass [M]^+^ = 532.1568 and observed mass [M]^+^ = 532.1564.

**4-Aminobutane CDP-choline (S10)** was synthesized accordingly from 4-azidobutane CDP-choline (**S8**) and isolated as a white solid (112 mg, 206 μmol, 80%). ^1^H NMR (600 MHz, D_2_O): δ 8.14 (d, *J* = 8.0 Hz, 1H), 6.29 (d, *J* = 8.0 Hz, 1H), 5.95 (d, *J* = 3.8 Hz, 1H), 4.42 (m, 2H), 4.25 (m, 6H), 4.19 (m, 1H), 3.72 to 3.68 (m, 2H), 3.57 to 3.53 (m, 2H), 3.18 (s, 6H), 3.11 (t, *J* = 7.5 Hz, 2H), and 2.20 to 2.18 (m, 2H); ^13^C NMR (151 MHz, D_2_O): δ 159.05, 148.31, 144.01, 95.18, 89.68, 83.09 (d, *J* = 9.0 Hz), 74.22, 69.03, 64.51, 63.35, 62.59, 61.36, 59.71 (d, *J* = 5.1 Hz), 51.93, 36.22, and 20.67; ^31^P NMR (162 MHz, D_2_O): δ −11.30 (d, *J* = 20.5 Hz) and −12.17 (d, *J* = 20.5 Hz); ESI-HRMS calculated mass [M]^+^ = 546.1725 and observed mass [M]^+^ = 546.1814.

#### General procedure for the synthesis of chloroacetamide- and bromoacetamide-CDP-choline analogs

CDP-choline analog **S9** or **S10** was dissolved in saturated aqueous NaHCO_3_ [CDP-choline (50 μl/mg)] and the corresponding *N*-hydroxysuccinimide ester of chloroacetic or bromoacetic acid (1.1 equivalent), was dissolved in little DMF, and was added to the stirring reaction. After completion (by TLC analysis), the solvents were removed under reduced pressure, and the residue was purified by reversed-phase column chromatography (Sep-Pak C18) applying an H_2_O/acetonitrile gradient (0 to 100% acetonitrile).

**Chloroacetamide-C3-CDP-choline (C3-Cl).** Yield: 30 mg, 63%, white wax. ^1^H NMR (600 MHz, D_2_O): δ 8.08 (d, *J* = 8.0 Hz, 1H), 6.19 (d, *J* = 7.9 Hz, 1H), 5.82 (d, *J* = 3.6 Hz, 1H), 4.31 to 4.19 (m, 6H), 4.12 to 4.07 (m, 1H), 4.03 (s, 2H), 3.61 to 3.55 (m, 2H), 3.38 to 3.31 (m, 2H), 3.26 (t, *J* = 6.6 Hz, 2H), 3.08 (s, 6H), and 2.01 to 1.93 (m, 2H); ^13^C NMR (151 MHz, D_2_O): δ 169.94, 159.04, 148.31, 144.05, 95.17, 89.64, 83.12, 74.24, 68.99, 64.51, 63.58, 63.02, 59.72, 59.69, 51.52, 42.19, 36.47, and 22.05; ^31^P NMR (243 MHz, D_2_O): δ −11.45 (d, *J* = 20.4 Hz) and −12.23 (d, *J* = 20.4 Hz); ESI-HRMS calculated mass [M]^+^ = 608.1284 and observed mass [M]^+^ = 608.1338.

**Chloroacetamide-C4-CDP-choline (C4-Cl).** Yield: 45 mg, 70%, white wax. ^1^H NMR (600 MHz, D_2_O): δ 7.85 (d, *J* = 8.0 Hz, 1H), 6.03 (d, *J* = 7.9 Hz, 1H), 5.92 (d, *J* = 3.6 Hz, 1H), 4.35 (s, 2H) 4.29 to 4.13 (m, 6H), 4.11 to 4.06 (m, 1H), 3.57 to 3.55 (m, 2H), 3.35 to 3.31 (m, 2H), 3.18 (t, *J* = 6.6 Hz, 2H), 3.08 (s, 6H), 2.39 to 1.33 (m, 2H), and 2.26 to 2.23 (m, 2H); ^13^C NMR (151 MHz, D_2_O): δ 166.04, 159.35, 148.48, 143.87, 95.23, 89.58, 83.15, 74.22, 68.78, 68.74, 63.73, 63.48, 62.43, 59.90, 59.78, 51.35, 42.30, 36.97, and 21.83; ^31^P NMR (243 MHz, D_2_O): δ −11.41 (d, *J* = 20.4 Hz) and −12.32 (d, *J* = 20.4 Hz); ESI-HRMS calculated mass [M]^+^ = 622.1440 and observed mass [M]^+^ = 622.1561.

**Bromoacetamide-C4-CDP-choline (C4-Br).** Yield: 33 mg, 69%, white wax. ^1^H NMR (600 MHz, D_2_O): δ 7.86 (d, *J* = 8.0 Hz, 1H), 6.04 (d, *J* = 7.9 Hz, 1H), 5.91 (d, *J* = 3.6 Hz, 1H), 4.30 (s, 2H) 4.27 to 4.15 (m, 6H), 4.13 to 4.08 (m, 1H), 3.58 to 3.54 (m, 2H), 3.36 to 3.32 (m, 2H), 3.23 (t, *J* = 6.6 Hz, 2H), 3.08 (s, 6H), 2.37 to 1.33 (m, 2H), and 2.26 to 2.23 (m, 2H); ^13^C NMR (151 MHz, D_2_O): δ 167.04, 159.34, 148.52, 143.95, 95.06, 89.34, 83.15, 74.32, 68.99, 68.75, 63.71, 63.58, 62.42, 59.92, 59.89, 51.45, 36.87, 28.12, and 21.73; ^31^P NMR (243 MHz, D_2_O): δ −11.22 (d, *J* = 20.4 Hz) and −12.17 (d, *J* = 20.4 Hz); ESI-HRMS calculated mass [M]^+^ = 666.0935 and observed mass [M]^+^ = 666.0889.

## Supplementary Material

aaz8041_Data_S1.xlsx

aaz8041_SM.pdf

## SUPPLEMENTARY MATERIALS

Supplementary material for this article is available at http://advances.sciencemag.org/cgi/content/full/6/20/eaaz8041/DC1

## Appendix

View/request a protocol for this paper from *Bio-protocol*.
