# Supplementary material for: Legionella effector AnkX displaces the switch II region for Rab1b phosphocholination
Source: Sci Adv. 2020 May 15;6(20):eaaz8041. doi: 10.1126/sciadv.aaz8041 (PMC7228754; doi:10.1126/sciadv.aaz8041)
Supplement: aaz8041_SM.pdf [file aaz8041_SM.pdf]

Science Advances
AAAS

advances.sciencemag.org/cgi/content/full/6/20/eaaz8041/DC1

Supplementary Materials for

Legionella effector AnkX displaces the switch II region for Rab1b phosphocholination

Stefan Ernst, Felix Ecker, Marietta S. Kaspers, Philipp Ochtrop, Christian Hedberg*, Michael Groll, Aymelt Itzen*

*Corresponding author. Email: christian.hedberg@umu.se (C.H.); a.itzen@uke.de (A.I.)

Published 15 May 2020, *Sci. Adv.* **6**, eaaz8041 (2020)
DOI: 10.1126/sciadv.aaz8041

The PDF file includes:

Figs. S1 to S10
Table S1
Legend for data S1
References

Other Supplementary Material for this manuscript includes the following:

(available at advances.sciencemag.org/cgi/content/full/6/20/eaaz8041/DC1)

Data S1

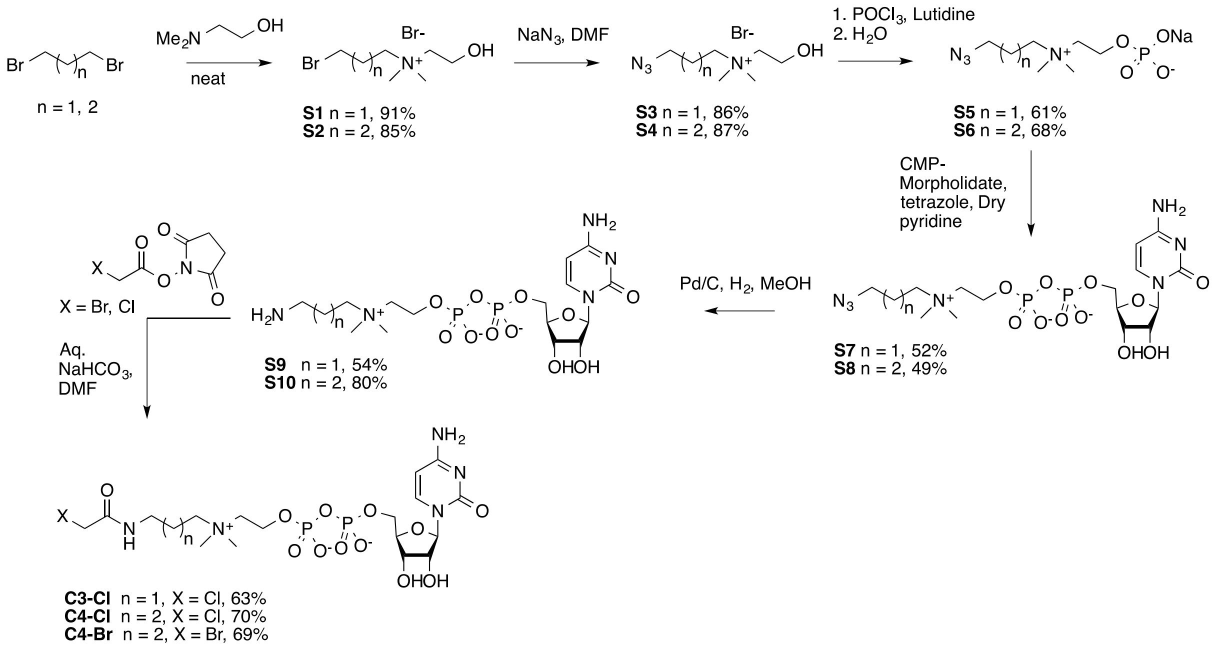

Fig. S1. Synthesis of CDP-choline derivatives. A large excess of dibromopropane (C3) or dibromobutane (C4) was reacted with N,N-dimethylaminoethanol, resulting in ammonium salts **S1** and **S2**, respectively. **S1** and **S2** were reacted with sodium azide in DMF, leading to azido-derivatives **S3** and **S4**. Direct phosphorylation with $POCl_3$ and lutidine provided mono-phosphates **S5** and **S6** after workup. **S5** and **S6** were coupled with CMP morpholidate via tetrazole activation under strictly anhydrous conditions, providing CDP-choline derivatives **S7** and **S8**. The azide-functionality of **S7** and **S8** were reduced by palladium on carbon / hydrogen. In this step, some material was lost due to absorption on the celite filtering aid. Resulting **S9** and **S10** from previous step were converted into the corresponding halo-acetamides by reaction with the corresponding NHS-esters under aq. conditions, thus providing the final compounds.

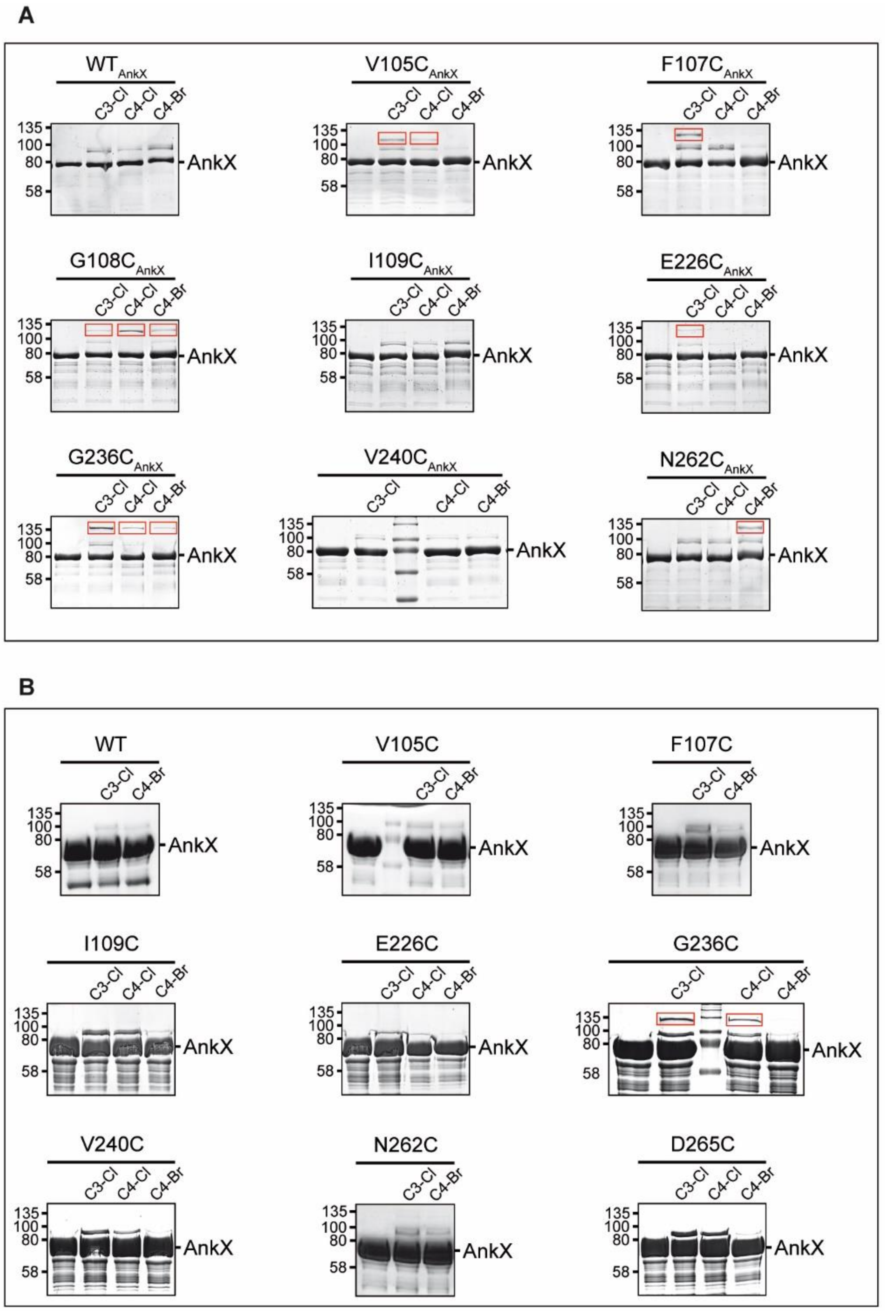

Fig. S2. Screening of AnkX cysteine mutants for ternary complex formation. (**A**) Direct approach. Screening of AnkX cysteine mutants (50 µM) and thiol-reactive CDP-choline analogues (1 mM) for their capability to produce a covalently linked complex with Rab1b (100 µM). Covalent complex formation was assessed by SDS-PAGE gel shift assay. The red rectangle indicates the band for the specific $AnkX_{Cys}$:PC:Rab1b complex. (**B**) Indirect approach. Screening of AnkX cysteine mutants (200 µM) and thiol-reactive CDP-choline analogues (1 mM) for their capability to produce a covalently linked complex with Rab1b (50 µM). Covalent complex formation was assessed by SDS-PAGE gel shift assay. The red rectangle indicates the band for the specific $AnkX_{Cys}$:PC:Rab1b complex.

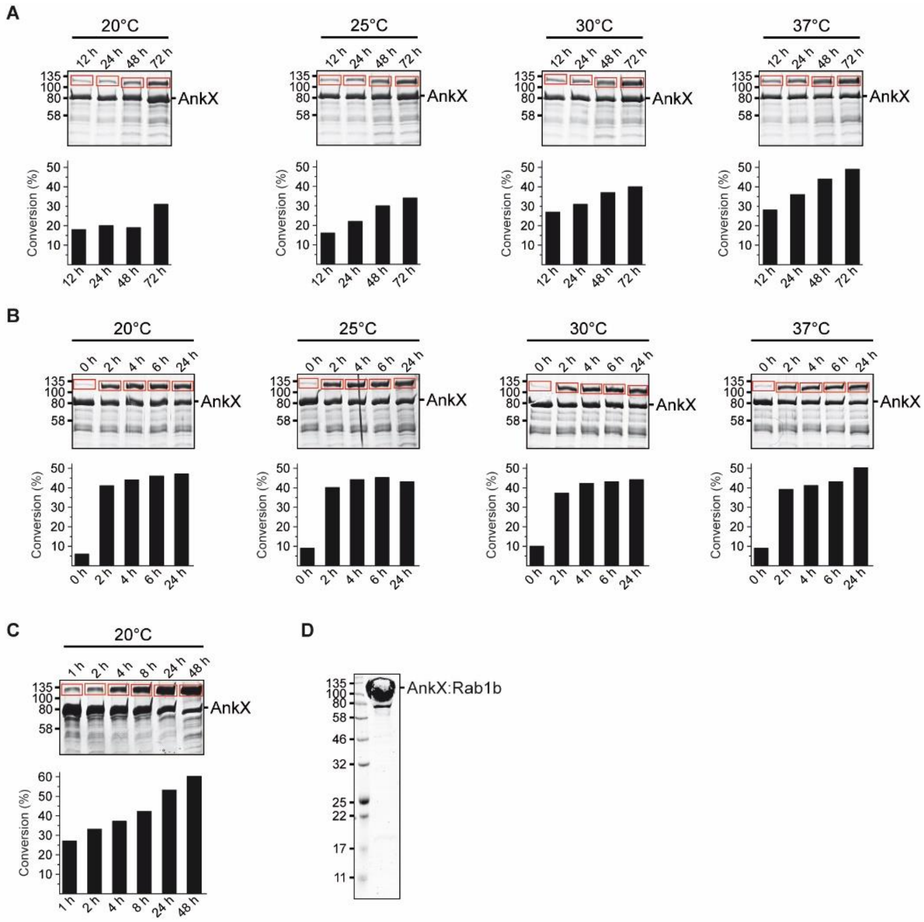

Fig. S3. Optimization of AnkX:Rab1b complex formation. (**A**) Optimization of the binary adduct formation between D265C$_{AnkX}$ (50 µM) and C3-Cl (1 mM) within the direct approach for different temperatures. Covalent complex formation between the binary conjugate (50 µM) and Rab1b (100 µM) was performed for 24 h at 20°C. Unspecific AnkX$_{D265C}$:PC:Rab1b complex formation could be prevented by purifying the binary adduct from excess CDP-choline derivative by buffer exchange before addition of Rab1b. The red rectangle indicates the band for the specific AnkX$_{D265C}$:PC:Rab1b complex. (**B**) Covalent complex formation between Rab1b (100 µM) and the binary adduct (50 µM) between AnkX$_{D265C}$ and C3-Cl within the direct approach at different temperatures. The binary conjugate between AnkX$_{D265C}$ (50 µM) and C3-Cl (1 mM) had been produced at 37°C for 48 h. Unspecific AnkX$_{D265C}$:PC:Rab1b complexes could be prevented by purifying the binary adduct from excess CDP-choline derivative by buffer exchange before addition of Rab1b. The red rectangle indicates the band for the specific AnkX$_{D265C}$:PC:Rab1b complex. (**C**) Covalent complex formation between AnkX$_{G108C}$ (200 µM) and Rab1b-PC (50 µM) at 20°C. Rab1b (50 µM) had been quantitatively modified with C3-Cl (1 mM) by catalytic amounts of wildtype AnkX (100 nM) at 20°C for 16 h. Unspecific AnkX$_{G108C}$:PC:Rab1b complexes could be prevented by replacing the cysteines C48, C84 and C172 with serines. The red rectangle indicates the band for the specific AnkX$_{G108C}$:PC:Rab1b complex. (**D**) SDS-PAGE analysis of AnkX$_{G108C}$:PC:Rab1b:GDP crystals. The absence of free Rab1b (20 kDa) is demonstrating the integrity of the covalent AnkX$_{G108C}$:PC:Rab1b:GDP complex.

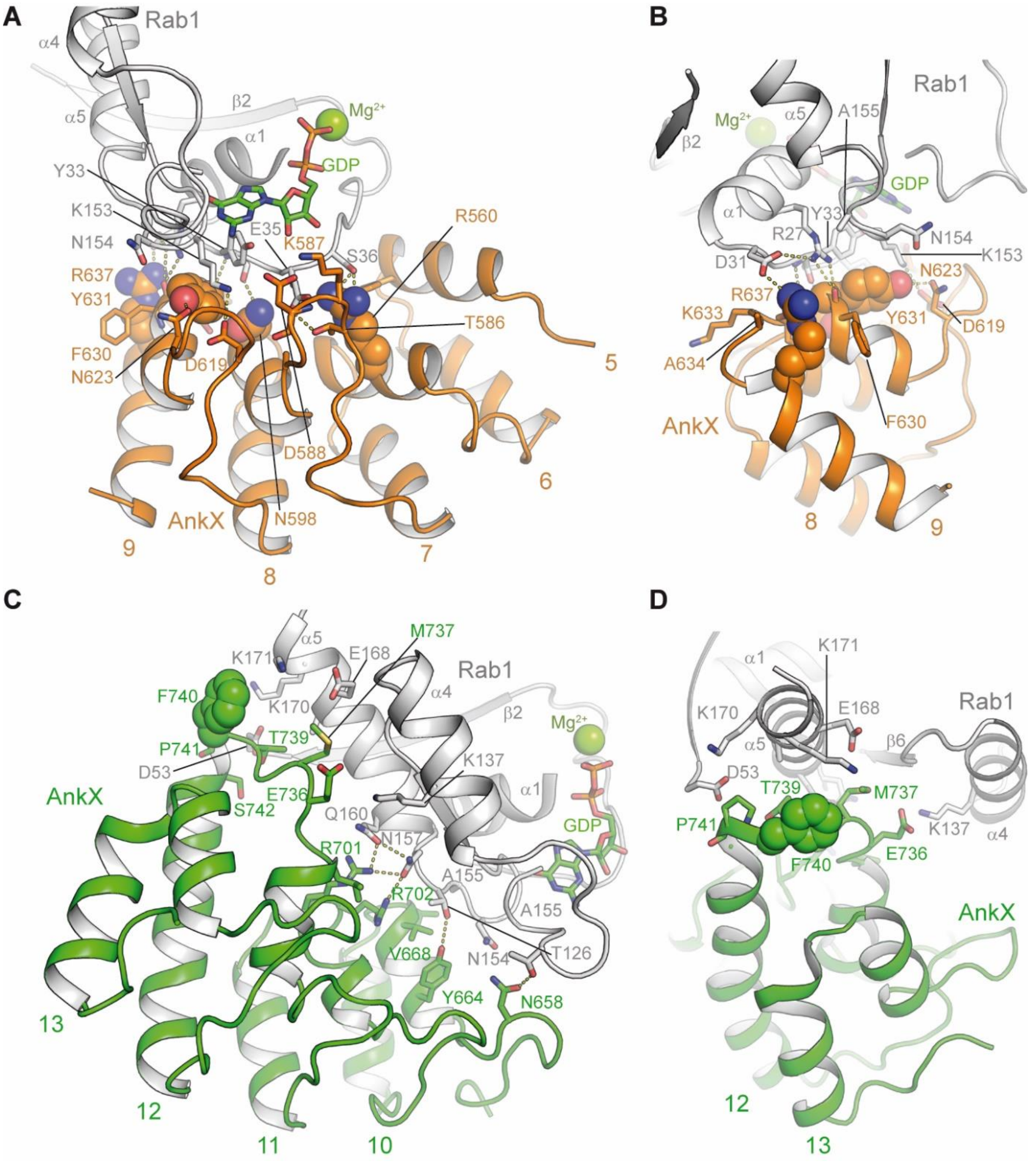

Fig. S4. Complex interface of Rab1b and the ankyrin repeats of AnkX. Rab1b is shown in grey, AnkX is coloured in orange and green for the ankyrin repeats 5-9 and 10-13, respectively. The colour code and numbering of the ankyrin repeats (integer numbers) matches Figure 3b. The putatively interacting amino acids have been identified using PDBsum *(35)*. AnkX residues, which mutation to alanine significantly affects Rab1b phosphocholination *in vitro*, are shown as spheres. (**A**) Front view of the AnkX:Rab1b interface showing only ankyrin repeats 5-9. (**B**) View of (A) rotated by 90°. (**C**) Front view of the AnkX:Rab1b interface showing only ankyrin repeats 10-13. (**D**) View of (C) rotated by 90°.

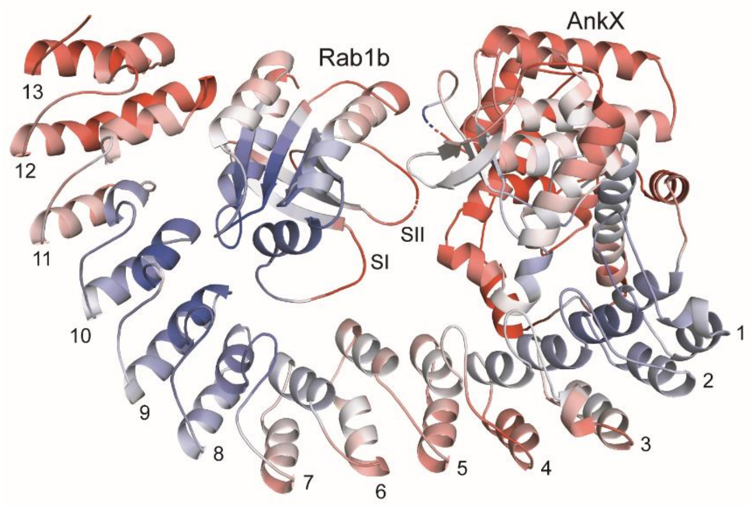

Fig. S5. The AnkX$_{G108C}$:PC:Rab1b:GDP complex colored by b-factors. The spectrum covers the range from 80 to 200 Å^2 (blue to white to red). The low b-factors of AnkX ankyrin repeats 8-11 are indicating low structural flexibility which may result from potent interactions with switch I and the C-terminus of Rab1b.

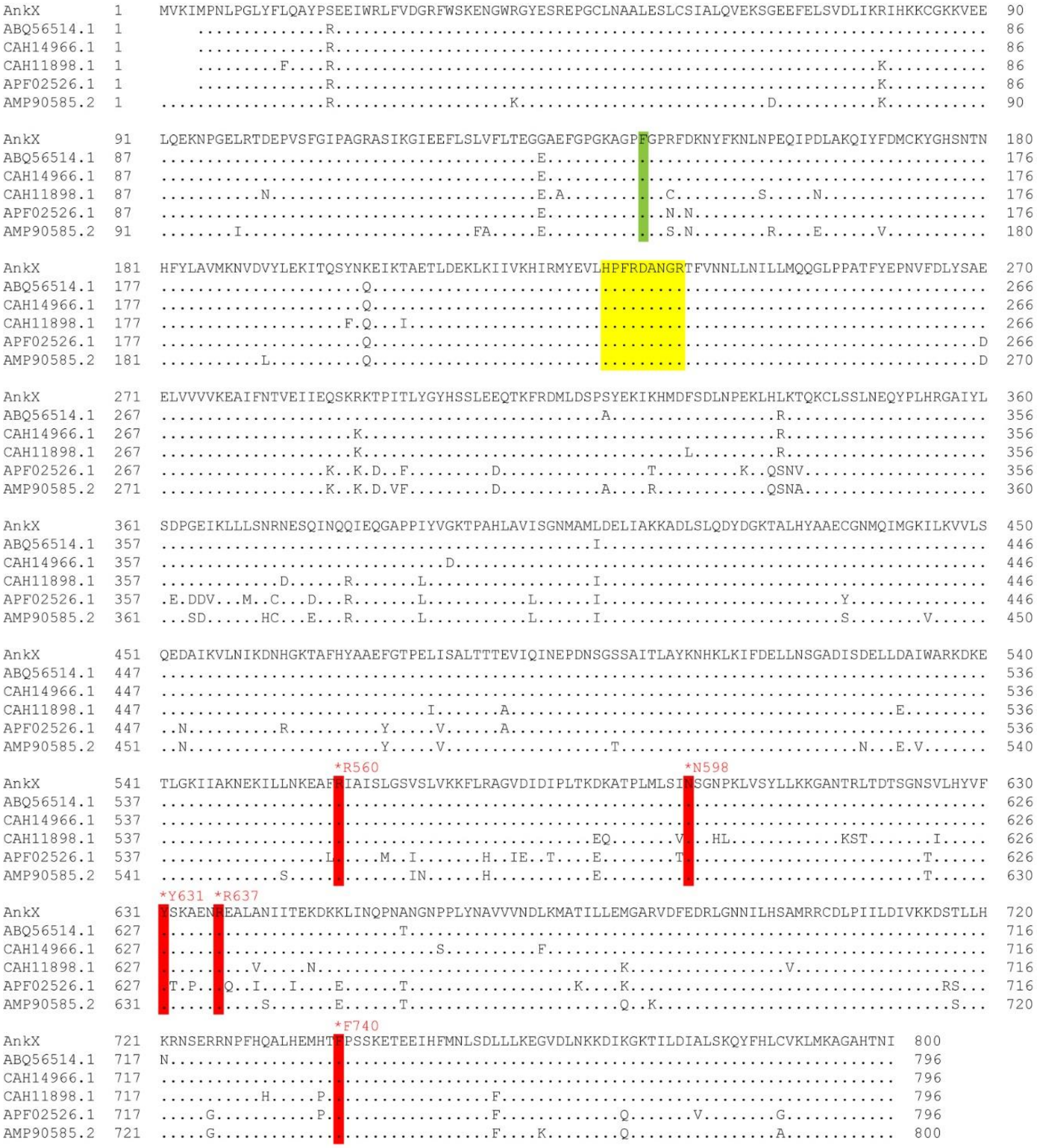

Accession codes

ABQ56514.1: Corby
CAH14966.1: Lens
CAH11898.1: Paris
APF02526.1: fraseri
AMP90585.2: pascullei

Fig. S6. Multiple sequence alignment of AnkX from different Legionella species. Dots indicate identical amino acids across different proteins. Amino acids, which mutations to alanine significantly affect AnkX activity, are highlighted in red. Green: F143; yellow: Fic-motif.

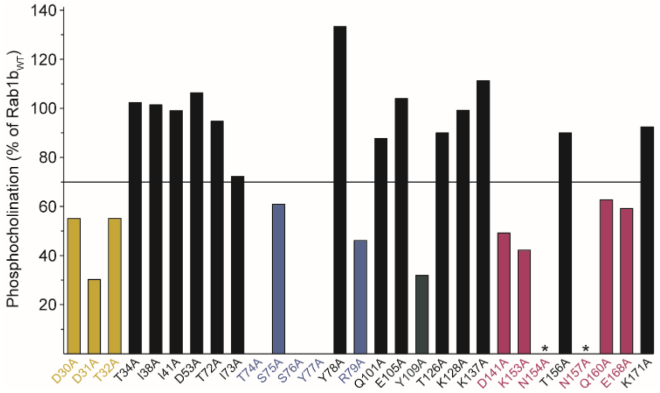

Fig. S7. AnkX-mediated phosphocholination of representative Rab1b alanine mutants within the AnkX-Rab1b binding interface. Rab1b alanine variants (1 µM) were modified with CDP-choline (50 µM) by catalytic amounts of AnkX$_{WT}$ (3.75 nM) for 1 h. The degree of phosphocholination was assessed with mass spectrometry and normalized to Rab1b$_{WT}$. For Rab1b alanine variants that were phosphocholinated to less than 70% of Rab1b$_{WT}$ catalytic efficiencies (k_{cat}/K_M) were determined using a time-resolved tryptophane fluorescence-based assay (see Fig. 3I). Blue: Rab1b switch I, magenta: Rab1b switch II, grey: α3 of Rab1b red: Rab1b C-Terminus. For Rab1b alanine variants marked with a star (*) no ion spectra could be recorded during mass spectrometry measurements.

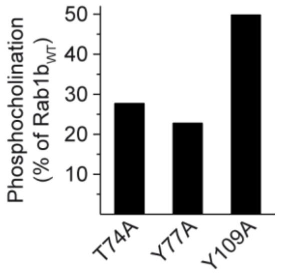

Fig. S8. Estimation of the catalytic efficiency of Rab1b$_{Y77A}$. Rab1b alanine variants (1 µM) were modified with CDP-choline (50 µM) by catalytic amounts of AnkX$_{WT}$ (3.75 nM) for 6 h and the degree of phosphocholination was assessed with mass spectrometry. The catalytic efficiency of Rab1b$_{Y77A}$ was estimated to be of similar scale as the catalytic efficiency of Rab1b$_{T74A}$.

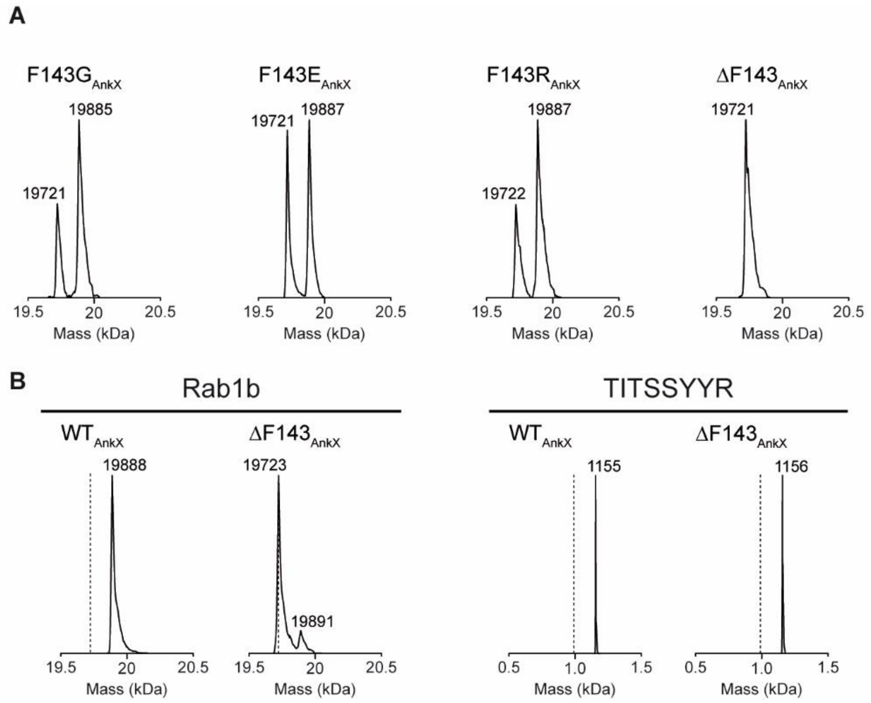

Fig. S9. Characterization of F143$_{AnkX}$ significance for catalysis. (**A**) Catalytic activity of F143$_{AnkX}$ mutants. Rab1b (5 µM) was modified with CDP-choline (1 mM) by catalytic amounts of the respective AnkX mutant (250 nM) for 8 h and the degree of phosphocholination was assessed with mass spectrometry. (**B**) Phosphocholination activity of ΔF143$_{AnkX}$ towards folded and unfolded AnkX substrates. Rab1b (50 µM) or the octapeptide TITSSYYR (50 µM) were incubated with CDP-choline (10 mM) and ΔF143$_{AnkX}$ (5 µM) or WT$_{AnkX}$ (5 µM) for 4 days. The degree of phosphocholination was assessed with mass spectrometry. The black dashed line indicates the mass of unmodified Rab1b (19729 Da) or octapeptide (990 Da), respectively.

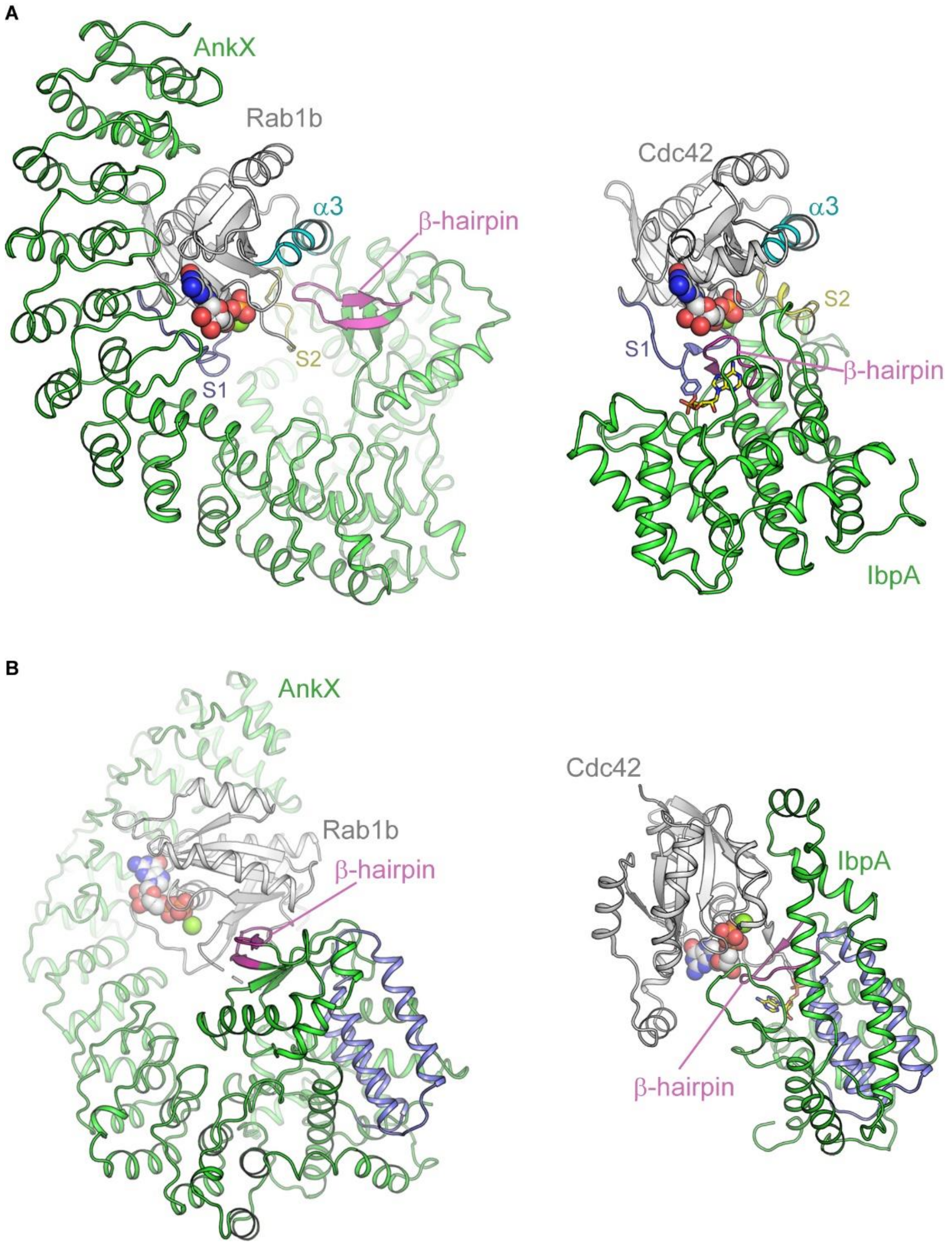

Fig. S10. Structural comparison of the AnkX:Rab1b and IbpA:Cdc42 complexes. (**A**) Structural superimposition of Rab1b (left) with Cdc42 (right) from their complexes with AnkX and IbpA, respectively. In the AnkX:Rab1b complex, the β-flap (= thorn) protrudes into a newly formed pocket between helix α3 and the switch II region (S2). In contrast, the β-flap of IbpA insert into the cleft between switch I (S1) and switch II. (**B**) Structural superimposition of AnkX (left) with IbpA (right) from their complexes with Rab1b and Cdc42, respectively. The structural basis for superimposition is the central core structure of Fic-enzymes consisting of three α-helices and the Fic-motif (highlighted in blue colour). Relative to Cdc42 in complex with IbpA, Rab1b is rotated by approximately 90° in the complex with AnkX. Spheres: bound guanine nucleotides.

Table S1. Crystallographic data collection and refinement statistics.

	**AnkX:Rab1b**
**Crystal parameters**	
Space group	C2
Cell constants	a= 177.4 Å, b= 68.5 Å, c= 137.4 Å<br>β= 98.9°
Subunits / AU[a]	2
**Data collection**	
Beam line	X06SA, SLS
Wavelength (Å)	1.0
Resolution range (Å)[b]	50 – 3.2 (3.3 – 3.2)
No. observed reflections	79,802
No. unique reflections[c]	25,684
Completeness (%)[b]	97.5 (98.1)
R_{merge} (%)[b, d]	4.5 (57.0)
I/σ (I)[b]	13.9 (2.3)
$CC_{1/2}$[b]	0.999 (0.813)
**Refinement (REFMAC5)**	
Resolution range (Å)	30 – 3.2
No. refl. working set	24,372
No. refl. test set	1,283
No. non hydrogen	7,557
No. of metals	1
No. of ligand atoms	28
R_{work} / R_{free} (%)[e]	24.7 / 28.8
r.m.s.d. bond (Å) / (°)[f]	0.002 / 1.2
Average B-factor ($Å^2$)	139
Ramachandran Plot (%)[g]	94.1 / 5.7 / 0.2
**PDB accession code**	**6SKU**

[a] Asymmetric unit

[b] The values in parentheses for resolution range, completeness, R_{merge}, I/σ (I) and $CC_{1/2}$ correspond to the highest resolution shell

[c] Data reduction was carried out with XDS and from a single crystal. Friedel pairs were treated as individual reflections

[d] $R_{merge}(I) = \Sigma_{hkl}\Sigma_j \mid I(hkl)_j - \langle I(hkl)\rangle \mid / \Sigma_{hkl} \Sigma_j I(hkl)_j$, where $I(hkl)_j$ is the jth measurement of the intensity of reflection hkl and $\langle I(hkl)\rangle$ is the average intensity

[e] $R = \Sigma_{hkl} \mid |F_{obs}| - |F_{calc}| \mid / \Sigma_{hkl} |F_{obs}|$, where R_{free} is calculated without a sigma cut off for a randomly chosen 5% of reflections, which were not used for structure refinement, and R_{work} is calculated for the remaining reflections

[f] Deviations from ideal bond lengths/angles

[g] Percentage of residues in favored region / allowed region / outlier region

Data S1. (separate file)

The spreadsheet contains a list of primers and constructs used in this study.

REFERENCES AND NOTES

1. R. K. Sherwood, C. R. Roy, A Rab-centric perspective of bacterial pathogen-occupied vacuoles. *Cell Host Microbe* **14**, 256–268 (2013).

2. S. Asrat, D. A. de Jesús, A. D. Hempstead, V. Ramabhadran, R. R. Isberg, Bacterial pathogen manipulation of host membrane trafficking. *Annu. Rev. Cell Dev. Biol.* **30**, 79–109 (2014).

3. I. Finsel, H. Hilbi, Formation of a pathogen vacuole according to *Legionella pneumophila*: How to kill one bird with many stones. *Cell. Microbiol.* **17**, 935–950 (2015).

4. J. Z. Qiu, Z. Q. Luo, *Legionella* and *Coxiella* effectors: Strength in diversity and activity. *Nat. Rev. Microbiol.* **15**, 591–605 (2017).

5. S. Mondino S. Schmidt, M. Rolando, P. Escoll, L. Gomez-Valero, C. Buchrieser, Legionnaires' disease: State of the art knowledge of pathogenesis mechanisms of *Legionella*. *Annu. Rev. Pathol.* **15**, 439–466 (2019).

6. J. Cherfils, M. Zeghouf, Regulation of small GTPases by GEFs, GAPs, and GDIs. *Physiol. Rev.* **93**, 269–309 (2013).

7. T. Murata, A. Delprato, A. Ingmundson, D. K. Toomre, D. G. Lambright, C. R. Roy, The *Legionella pneumophila* effector protein DrrA is a Rab1 guanine nucleotide-exchange factor. *Nat. Cell Biol.* **8**, 971–977 (2006).

8. M. P. Machner, R. R. Isberg, Targeting of host Rab GTPase function by the intravacuolar pathogen *Legionella pneumophila*. *Dev. Cell* **11**, 47–56 (2006).

9. A. Ingmundson, A. Delprato, D. G. Lambright, C. R. Roy, *Legionella pneumophila* proteins that regulate Rab1 membrane cycling. *Nature* **450**, 365–369 (2007).

10. M. P. Müller, H. Peters, J. Blümer, W. Blankenfeldt, R. S. Goody, A. Itzen, The *Legionella* effector protein DrrA AMPylates the membrane traffic regulator Rab1b. *Science* **329**, 946–949 (2010).

11. Y. H. Tan, Z. Q. Luo, *Legionella pneumophila* SidD is a deAMPylase that modifies Rab1. *Nature* **475**, 506–509 (2011).

12. M. R. Neunuebel, Y. Chen, A. H. Gaspar, P. S. Backlund Jr., A. Yergey, M. P. Machner, De-AMPylation of the small GTPase Rab1 by the pathogen *Legionella pneumophila*. *Science* **333**, 453–456 (2011).

13. S. Mukherjee, X. Liu, K. Arasaki, J. McDonough, J. E. Galán, C. R. Roy, Modulation of Rab GTPase function by a protein phosphocholine transferase. *Nature* **477**, 103–106 (2011).

14. P. R. Goody, K. Heller, L. K. Oesterlin, M. P. Müller, A. Itzen, R. S. Goody, Reversible phosphocholination of Rab proteins by *Legionella pneumophila* effector proteins. *EMBO J.* **31**, 1774–1784 (2012).

15. K. Gavriljuk, J. Schartner, H. Seidel, C. Dickhut, R. P. Zahedi, C. Hedberg, C. Kötting, K. Gerwert, Unraveling the phosphocholination mechanism of the *Legionella pneumophila* enzyme AnkX. *Biochemistry* **55**, 4375–4385 (2016).

16. Y. H. Tan, R. J. Arnold, Z. Q. Luo, *Legionella pneumophila* regulates the small GTPase Rab1 activity by reversible phosphorylcholination. *Proc. Natl. Acad. Sci. U.S.A.* **108**, 21212–21217 (2011).

17. T. M. Lovell, R. J. Woods, D. J. Butlin, K. J. Brayley, I. T. Manyonda, J. Jarvis, S. Howell, P. J. Lowry, Identification of a novel mammalian post-translational modification, phosphocholine, on placental secretory polypeptides. *J. Mol. Endocrinol.* **39**, 189–198 (2007).

18. X. X. Pan, A. Lührmann, A. Satoh, M. A. Laskowski-Arce, C. R. Roy, Ankyrin repeat proteins comprise a diverse family of bacterial type IV effectors. *Science* **320**, 1651–1654 (2008).

19. V. Campanacci, S. Mukherjee, C. R. Roy, J. Cherfils, Structure of the *Legionella* effector AnkX reveals the mechanism of phosphocholine transfer by the FIC domain. *EMBO J.* **32**, 1469–1477 (2013).

20. L. K. Oesterlin, R. S. Goody, A. Itzen, Posttranslational modifications of Rab proteins cause effective displacement of GDP dissociation inhibitor. *Proc. Natl. Acad. Sci. U.S.A.* **109**, 5621–5626 (2012).

21. Y. Chen, I. Tascón, M. R. Neunuebel, C. Pallara, J. Brady, L. N. Kinch, J. Fernández-Recio, A. L. Rojas, M. P. Machner, A. Hierro, Structural basis for Rab1 de-AMPylation by the *Legionella pneumophila* effector SidD. *PLOS Pathog.* **9**, e1003382 (2013).

22. P. Ochtrop, S. Ernst, A. Itzen, C. Hedberg, Exploring the substrate scope of the bacterial phosphocholine transferase AnkX for versatile protein functionalization. *Chembiochem* **20**, 2336–2340 (2019).

23. K. Heller, P. Ochtrop, M. F. Albers, F. B. Zauner, A. Itzen, C. Hedberg, Covalent protein labeling by enzymatic phosphocholination. *Angew. Chem. Int. Ed. Engl.* **54**, 10327–10330 (2015).

24. S. Eathiraj, X. Pan, C. Ritacco, D. G. Lambright, Structural basis of family-wide Rab GTPase recognition by rabenosyn-5. *Nature* **436**, 415–419 (2005).

25. J. Xiao, C. A. Worby, S. Mattoo, B. Sankaran, J. E. Dixon, Structural basis of Fic-mediated adenylylation. *Nat. Struct. Mol. Biol.* **17**, 1004–1010 (2010).

26. M. P. Müller, A. V. Shkumatov, L. K. Oesterlin, S. Schoebel, P. R. Goody, R. S. Goody, A. Itzen, Characterization of enzymes from *Legionella pneumophila* involved in reversible adenylylation of Rab1 protein. *J. Biol. Chem.* **287**, 35036–35046 (2012).

27. Y. W. Wu, L. K. Oesterlin, K. T. Tan, H. Waldmann, K. Alexandrov, R. S. Goody, Membrane targeting mechanism of Rab GTPases elucidated by semisynthetic protein probes. *Nat. Chem. Biol.* **6**, 534–540 (2010).

28. S. C. Allgood, B. P. Romero Dueñas, R. R. Noll, C. Pike, S. Lein, M. R. Neunuebel, *Legionella* effector AnkX disrupts host cell endocytic recycling in a phosphocholination-dependent manner. *Front. Cell. Infect. Microbiol.* **7**, 397 (2017).

29. S. Schoebel, L. K. Oesterlin, W. Blankenfeldt, R. S. Goody, A. Itzen, RabGDI displacement by DrrA from *Legionella* is a consequence of its guanine nucleotide exchange activity. *Mol. Cell* **36**, 1060–1072 (2009).

30. W. Kabsch, *XDS*. *Acta Crystallogr. D Biol. Crystallogr.* **66**, 125–132 (2010).

31. P. D. Adams, P. V. Afonine, G. Bunkóczi, V. B. Chen, I. W. Davis, N. Echols, J. J. Headd, L. W. Hung, G. J. Kapral, R. W. Grosse-Kunstleve, A. J. McCoy, N. W. Moriarty, R. Oeffner, R. J. Read, D. C. Richardson, J. S. Richardson, T. C. Terwilliger, P. H. Zwart, PHENIX: A comprehensive python-based system for macromolecular structure solution. *Acta Crystallogr. D Biol. Crystallogr.* **66**, 213–221 (2010).

32. P. Emsley, B. Lohkamp, W. G. Scott, K. Cowtan, Features and development of Coot. *Acta Crystallogr. D Biol. Crystallogr.* **66**, 486–501 (2010).

33. G. N. Murshudov, P. Skubák, A. A. Lebedev, N. S. Pannu, R. A. Steiner, R. A. Nicholls, M. D. Winn, F. Long, A. A. Vagin, REFMAC5 for the refinement of macromolecular crystal structures. *Acta Crystallogr. D Biol. Crystallogr.* **67**, 355–367 (2011).

34. Z. Zhang, A. G. Marshall, A universal algorithm for fast and automated charge state deconvolution of electrospray mass-to-charge ratio spectra. *J. Am. Soc. Mass Spectrom.* **9**, 225–233 (1998).

35. R. A. Laskowski, J. Jabłońska, L. Pravda, R. S. Vařeková, J. M. Thornton, PDBsum: Structural summaries of PDB entries. *Protein Sci.* **27**, 129–134 (2018).
